# Mitochondria Need Their Sleep: Redox, Bioenergetics, and Temperature Regulation of Circadian Rhythms and the Role of Cysteine-Mediated Redox Signaling, Uncoupling Proteins, and Substrate Cycles

**DOI:** 10.3390/antiox12030674

**Published:** 2023-03-09

**Authors:** Richard B. Richardson, Ryan J. Mailloux

**Affiliations:** 1 Radiobiology and Health, Canadian Nuclear Laboratories (CNL), Chalk River, ON K0J 1J0, Canada; 2 McGill Medical Physics Unit, Cedars Cancer Centre—Glen Site, McGill University, Montreal, QC H4A 3J1, Canada; 3 School of Human Nutrition, Faculty of Agricultural and Environmental Sciences, McGill University, Sainte-Anne-de-Bellevue, QC H9X 3V9, Canada; ryan.mailloux@mcgill.ca

**Keywords:** bioenergetics, circadian, cysteine oxoforms, mitochondria, nuclear, oxidative stress, redoxome, substrate cycles, temperature, uncoupling

## Abstract

**Highlights:**

**What are the main findings?**

- Hypothesis: Redox, bioenergetic and temperature regulation is critical in maintaining cellular circadian rhythms; wakefulness is mainly “nucleorestorative” and sleep is mainly “mitorestorative”.
- Wakefulness: High metabolic rate induces oxidative stress and redox imbalance.
- Sleep: Fusion remodels mitochondria and the cellular redox balance is restored.
- Sleep: Mitochondria aid activation of rapid immune, inflammatory and heat shock responses.

**What is the implication of the main findings?**

- Sleep-wake cycling: Provides insights into the role of cysteine-mediated redox signaling, uncoupling and substrate cycles.
- Disorders of human development and aging: Perturbations of circadian tripartite-interactome signaling and mitochondrial-nuclear coregulation are implicated.

**Abstract:**

Although circadian biorhythms of mitochondria and cells are highly conserved and crucial for the well-being of complex animals, there is a paucity of studies on the reciprocal interactions between oxidative stress, redox modifications, metabolism, thermoregulation, and other major oscillatory physiological processes. To address this limitation, we hypothesize that circadian/ultradian interaction of the redoxome, bioenergetics, and temperature signaling strongly determine the differential activities of the sleep–wake cycling of mammalians and birds. Posttranslational modifications of proteins by reversible cysteine oxoforms, S-glutathionylation and S-nitrosylation are shown to play a major role in regulating mitochondrial reactive oxygen species production, protein activity, respiration, and metabolomics. Nuclear DNA repair and cellular protein synthesis are maximized during the wake phase, whereas the redoxome is restored and mitochondrial remodeling is maximized during sleep. Hence, our analysis reveals that wakefulness is more protective and restorative to the nucleus (nucleorestorative), whereas sleep is more protective and restorative to mitochondria (mitorestorative). The “redox–bioenergetics–temperature and differential mitochondrial–nuclear regulatory hypothesis” adds to the understanding of mitochondrial respiratory uncoupling, substrate cycling control and hibernation. Similarly, this hypothesis explains how the oscillatory redox–bioenergetics–temperature–regulated sleep–wake states, when perturbed by mitochondrial interactome disturbances, influence the pathogenesis of aging, cancer, spaceflight health effects, sudden infant death syndrome, and diseases of the metabolism and nervous system.

## 1. Introduction

The differential activities of sleep–wake cycling are known to be necessary and important for the well-being of complex animals, such as mammals, birds, and reptiles [1,2]. Although there are individual reports on the regulation of circadian rhythms by reactive oxygen/nitrogen/sulfur species (ROS/RNS/RSS), the redoxome, metabolomics, bioenergetics, and the thermal state during sleep–wake cycling (or resting–active phases), there is little published on their significant interaction; therefore, a clearer understanding of these reciprocity issues warrants attention, especially the changes wrought by aging and disease. Considering aging alone, the “free radical theory of aging” posits that species of animals with higher metabolic rates produce more oxidative stress, resulting in a shorter life span [3]; however, this relationship does not hold for all species [4]. Another explanation of longevity is the earlier “rate of living hypothesis” that states that the lower the metabolic rate of a species, the longer its life expectancy [5,6]. Mitochondria also have a major role in the aging process. They not only affect the metabolic rate but also the production and control of oxidative stress [3,4]. Therefore, even aging alone is determined not by one but by several interactive physiologic effects.

Free-radical, metabolic, and mitochondrial processes are generally in dynamic equilibrium rather than in a steady state. Therefore, life expectancy hypotheses involving these processes are complicated by diurnal and seasonal variations. Endothermic mammals and birds with smaller mass generally have a longer maximum life span and higher mass-specific metabolic rate, but they also sleep longer [7]. Mice sleep about four times longer than elephants, which sleep for only 3.5 h or less daily. Human resting energy expenditure is highest in the afternoon and evening and reduced by 10–30% at its lowest during sleep [8,9]. Another circadian parameter, ROS levels, was measured in leukocytes of adults aged 21–25 years old and found to significantly peak at 18:00 and again at 3:00 [10]. There is no question that eukaryote cells have extensively evolved with the bioenergetic advantages of atmospheric oxygen and the cytotoxicity of ROS, especially mitochondrial ROS; so much so that ROS and redox-signaling molecules are essential facets of multicellular organisms. Therefore, the diurnal oscillatory nature of ROS is critical when considering redox-controlled biological mechanisms associated with the sleep–wake metabolic states of mammals and birds.

In general, metabolic rate scales with body size and temperature. The resting metabolic rate per gram of body weight of a mouse is about sevenfold greater than in humans. One estimate of the total energy expenditure of a sedentary man suggests that a 10th is accounted for by physical activity, a 10th is adaptive, facultative thermogenesis (due to the cold or dietary intake), and eight 10ths are due to the basal/resting metabolic rate [11]. About two-thirds of the resting metabolic rate is dedicated to homeothermy, that is, to maintaining a stable core temperature. Therefore, homeothermy accounts for about 50% of the total energy expenditure in humans and a similar percentage in mice [11]. Thermoregulation in mammals and birds keeps their internal temperature steady, within a few degrees Celsius [12]. This internal temperature, generally higher than that of their surroundings, requires both physiologic and behavioral mechanisms to keep the temperature within a narrow range. It has been estimated from patients with fever that the metabolic rate increases by 13%/°C [13]. The reason for this elevated and finely tuned body temperature in endothermic/homoeothermic species is unknown [12]. The core and brain temperatures of humans (children and adults) drop during sleep by about 1 °C, lowest 3 h before wakening, and rise during the day to a peak in the evening [14]. A crucial aspect of the redox–bioenergetics–temperature and differential mitochondrial–nuclear regulatory hypothesis we develop is that the lowering of core temperature during sleep increases oxidative stress in mitochondria [15], necessitating uncoupling proteins (UCPs) to lower ROS levels and raise heat production.

It is recognized that the circadian rhythms of physiological processes at all organizational levels in the body are controlled by central and peripheral “clocks,” with the “redox clock” being one of the latter [16]. Four-fifths of protein-coding genes, in at least one of 64 tissues and brain regions from baboons, exhibited 24 h rhythms in gene expression [17]. In mammals, the central circadian clock is the suprachiasmatic nucleus (SCN) of the hypothalamus that genetically oscillates and is primarily influenced by light. The mammalian target of rapamycin (mTOR) has a key role in the entrainment of the SCN. Downstream targets of mTOR complex 1 (mTORC1) promote the synthesis of both protein and glutathione [18]. The SCN also entrains slave oscillators in extra-SCN brain regions and peripheral tissues. However, every cell in the body has its own reciprocal circadian rhythm–redox state timekeeping and even the SCN can be modulated by the redox state downstream of the free radical, nitric oxide (^•^NO) [19].

Oxidative stress arises from redox imbalance or bias between antioxidants and pro-oxidants. Over 90% of mammalian oxygen consumption is by mitochondria, which are also the major cellular source of ROS, such as superoxide radicals O_2_^•−^, hydroxyl radicals ^•^OH, hydrogen peroxide H_2_O_2_ and the peroxide functional group ROOR [20,21]. RNS include ^•^NO and the powerful oxidant peroxynitrite ONOO^−^. Oxygen is normally reduced via the mitochondrial electron transport chain (ETC) by four electrons to water when they arrive at cytochrome C oxidase, the terminal ETC complex, also known as complex IV (CIV). Alternatively, oxygen is terminally reduced by one, two, or three electrons, respectively, via mitochondrial redox carriers CI, CII, and CIII yielding ROS: O_2_^•−^, H_2_O_2_, or ^•^OH, respectively. These mitochondrial ROS are generated by <2% of ETC electrons that leak from the ETC, mostly via CI, and interact with oxygen not consumed by mitochondrial respiration [22].

Enzymatic antioxidants are a principal means of counteracting this oxidative stress. These enzymes include catalase, glutathione peroxidase (GPX), peroxiredoxin (PRX), superoxide dismutase (SOD), and thioredoxin (TRX). Oxidative stress is also lessened by nonenzymatic antioxidants of glutathione (GSH, γ-glutamyl-cysteinyl-glycine), melatonin, uric acid, vitamins C, and vitamin E. Enzymatic antioxidants in humans usually peak during the light phase (e.g., catalase, GPX, PRX, and SOD), whereas most nonenzymatic antioxidants peak during the dark phase (melatonin, vitamins C and E, GSH, but not uric acid) [23,24,25]. The understanding of human circadian trends of enzymatic and nonenzymatic antioxidants is augmented by rodent and other animal studies, taking account of species differences and that animals such as rodents have a nocturnal wake state, in contrast to the diurnal activity of humans [26].

Two important redox processes that are protective to mitochondria (i.e., mitoprotective) are anion carriers and posttranslational modifications of protein thiols [27]. The mitochondrial anion carrier protein family, located in the mitochondrial inner membrane, includes the subfamilies of the UCPs and adenine nucleotide translocase (ANT). The latter catalyzes the exchange of the adenosine diphosphate (ADP) anion for adenosine triphosphate (ATP). Anionic fatty acids, superoxide, and peroxidation products transfer across the mitochondrial inner membrane via UCPs and ANT activating the inducible proton leak [28]. The mitochondrial coupling efficiency, calculated as the ATP generated by oxidative phosphorylation per molecule of oxygen consumed, is reduced by proton leakage, and some of the proton motive force (membrane potential and proton gradient) is converted to heat. Mitochondrial proton leaks and uncoupling of mitochondrial electron transport from phosphorylation are the principal means of counteracting oxidative stress by reducing the mitochondrial inner membrane potential that lessens the electron leakage along with ROS generation [29,30]. Thiol-based and non-thiol protein posttranslational modifiers are involved in circadian regulation. Some of these modifications are reversible, including phosphorylation, glycosylation, ubiquitination, methylation, and acetylation. Our focus is on the reversible cysteine oxoforms—S-glutathionylation (-S-SG-) and S-nitrosation (also referred to as S-nitrosylation (-S-NO))—that provide proteins with redox respiratory protective measures to minimize oxidative and nitrosative stress.

**Figure 1:**
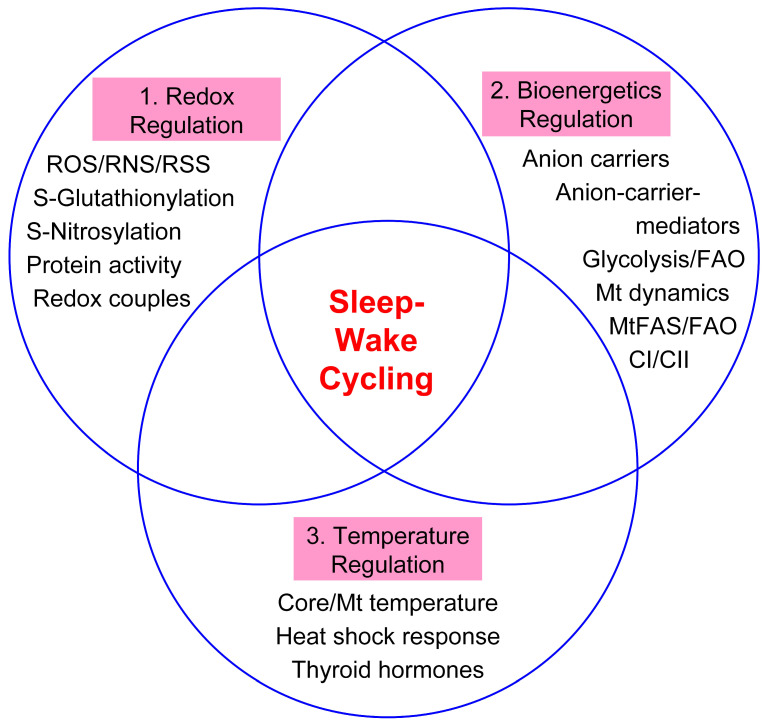
Interconnectedness of redoxome, bioenergetics and thermal regulation. The various cellular and mitochondrial (Mt) activities associated with the tripartite-interactome components, such as complexes I and II (CI/CII), fatty-acid oxidation (FAO), fatty-acid synthesis (FAS), and reactive oxygen/nitrogen/sulfur species (ROS/RNS/RSS), are also listed.

On examination of the redoxome, bioenergetics, and thermal regulatory processes in humans, we hypothesize that the interactome, metabolic, and physiological circadian oscillations are strongly regulated by cellular cysteine-mediated posttranslational modifications of proteins, mitochondrial respiratory uncoupling, and temperature (Figure 1). The case is made that these spatiotemporal cycles of the redoxome, bioenergetics and temperature promote a wake phase that is more protective and restorative to the nucleus, which we term “nucleorestorative,” whereas sleep is more protective and restorative to mitochondria, hence “mitorestorative.” Following the introductory first section, the second section describes circadian posttranslational modifications and redox couples comprising electron donor and electron acceptor molecules. The third section focuses on the primary processes controlling mitochondrial bioenergetics, such as anion carriers (including UCPs), anion carrier mediators (such as thyroid hormones and melatonin), and the temporal separation of substrate or futile cycles. The fourth section describes how core temperature and other stressors affect mitochondrial heat shock response, oxidative stress, and temperature-dependent rhythms. The fifth section gives the implications of the redox–bioenergetics–temperature and differential mitochondrial–nuclear regulatory hypothesis in terms of uncoupling theories, childhood development, aging and related-diseases, hibernation in animals, and the effects of space radiation. In the sixth section, the implications of the hypothesis are given in view of sleep theories, followed by the concluding final section.

## 2. Protein Posttranslational Modifications, Redox Couples, and Circadian Rhythms

### 2.1. Introduction

Eighty-two percent of the primate protein-coding genome (~18,000 genes) assessed in 64 tissues displayed circadian gene expression common to at least one other tissue [17]. Rhythmic gene expression peaked in the early afternoon (~11,000 genes), with a trough around midnight (~700 genes). Although there is a considerable universally expressed diurnal transcriptome in all tissues, circadian gene expression is generally tissue-, organelle- and study-specific. For example, only 8% of the total proteins of mouse liver (575 of 6780 proteins) were diurnal-oscillating [31]. A separate study reported a far larger percentage: ~38% of the mitochondrial proteome (223 of 590 proteins) oscillates in mouse liver [32]. The predominant peak abundance of cycling mitochondrial proteins was during the early light phase, with a lesser peak 12 h later. Posttranslational modification of proteins, especially phosphorylation and acetylation, are important circadian regulators, but for brevity, these will not be detailed. That said, global quantification of the mouse liver proteome has shown that phosphorylation is an important circadian regulator of the metabolism [33]. Two-thirds of acetylation sites in the liver mitochondria from mice were localized to proteins. Of note is the role of the mitochondrial sirtuin 3 (*SIRT3*) gene in coordinating posttranslational acetylation modification, as NAD^+^-dependent sirtuins in general are prominent regulators of diverse pathways, including circadian regulation of the cellular metabolism and mitochondrial maintenance [34].

In this section, the focus is on oxidative eustress and redox-based posttranslational modifications of proteins by reversible cysteine oxoforms, including S-glutathionylation (-S-SG-), S-nitrosylation (-S-NO), S-sulfenylation (-S-OH) and disulfide bonds (-S-S-). In the presence of oxidative distress, such as that caused by GSH depletion, cellular S-glutathionylation can become hyperoxidized and irreversible by the thiol oxidation route, with cysteine residues oxidized to sulfenic (RSOH), sulfinic (RSO_2_H) and finally sulfonic (RSO_3_H) acids [35]. There are normal diurnal variations in oxidative eustress. A universal feature of aerobic eukaryotes is ~24 h circadian timekeeping by posttranslational modifications that control oscillatory cellular and mitochondrial redox states [36]. The cysteine oxoforms of proteins that have circadian entrainment and reside in mitochondria are of especial interest. Lastly, the reduced-to-oxidized ratios of crucial redox couples are reviewed as these indicators of redox status are usually circadian-regulated.

### 2.2. Cellular and Mitochondrial S-Nitrosylation and Circadian Oscillations

S-nitrosylation is a circadian oxidative modification of a thiol (-SH) by the reversible covalent bonding to a nitric oxide group (-NO) to form S-nitrosothiols (-S-NO) in a target protein. Protein S-nitrosylation is generally short-lived: denitrosylated proteins are TRX and GSH. On the one hand, excess GSH can cause nitrosylated cysteines to spontaneously denitrosylate [37], and on the other hand, excess ^•^NO produced by nitric acid synthase (NOS) causes GSH depletion, as induced in rat midbrain cultures [38]. The main physiological influence of the radical-gas ^•^NO, with a short half-life of a few seconds, is via protein translation by hypo- or hyper-S-nitrosylation, increasing the activity of specific protein targets such as hypoxia-inducible factor 1 (HIF-1) or dynamin-related protein 1 (DRP1) [39]. The ^•^NO stress response is a secondary effect. For example, ^•^NO-dependent S-nitrosylation is a protective modification that reduces ROS by inhibiting mitochondrial CI activity [40].

S-nitrosylation mediated by ^•^NO appears to be overrepresented in mitochondrial proteins compared to the whole-cell proteome. Doulias et al. [41] found only 119 S-nitrosylated proteins in each of the heart and brain, with 56% and 25% of their respective proteins localized to mitochondria. Similarly, of the 123–340 S-nitrosylated proteins found in kidney, liver, lung, and thymus, 20–25% of their S-nitrosoproteomes consisted of mitochondrial proteins. S-nitrosoproteome mainly influences the cellular metabolism and mitochondrial bioenergetics, especially β-oxidation of fatty acids. Endogenous ^•^NO production from NOS undergoes circadian variation (and ultradian changes [42]) reciprocally regulating and regulated by biological clocks [43]. ^•^NO and NOS contribute significantly to daily sleep pressure or homeostatic sleep. Peak ^•^NO production varies with the rodent tissue under study, for example, endothelial NOS provides a redox-signaling pathway with ^•^NO in serotonergic neurons at maximum during rapid eye moment (REM, composed of one stage) sleep phase, rather than during non-REM (NREM, of three stages) sleep phase [42,44].

### 2.3. Cellular S-Glutathionylation

S-glutathionylation is the reversible binding of GSH via a disulfide bond to thiolate anions of cysteines in target proteins. Deglutathionylation is performed by the major cysteine source GSH or by catalysts glutaredoxin (GRX) or sulfiredoxin (SRX) [45]. Alternatively, S-glutathionylation can be catalyzed by glutathione synthetase transferase π (GSTπ), which is a detoxification enzyme with a turnover time within 2–3 h following ROS/RNS-related stress [46]. Protein S-glutathionylation in general inhibits protein function and protects against both oxidative distress and irreversible oxidation [47,48].

There are several studies on protein targets for S-glutathionylation for various cell types. Fratelli et al. [47] identified approximately 300 S-glutathionylation targets of diamide- and hydrogen peroxide-induced oxidatively stressed T cells. Targets included cytoskeletal proteins, the mitochondrial homologue of the heat stress protein family, HSP70 (i.e., HSP70-9B mortalin-2), glycolysis (e.g., the penultimate step in glycolysis, enolase), and the synthesis of fatty acids and amino acids. A review by Townsend [49] deemed that cellular S-glutathionylation corresponds to a small fraction of the proteome, specific to six clusters of proteins, which are cytoskeletal, metabolism and energy, kinase/phosphatase signaling pathways, calcium homeostasis, protein folding/stability, and redox homeostasis. Duan et al. [50] identified 2494 S-glutathionylation-modified sites on 1276 proteins in virus-transformed macrophage-like cells (from mice) exposed to moderate nanoparticle-induced oxidative stress. Depending on the specific treatment, 27–56% of the 2494 S-glutathionylation-modified sites were significantly altered, particularly those sites that regulated cellular protein synthesis, protein stability, and phagocytosis. In contrast, at higher ROS levels, proteins involved in intermediary metabolism, cell adhesion and oxidative stress response pathways were preferentially posttranslationally modified. These three S-glutathionylation cellular studies show that target proteins are dependent not only on the cell type analyzed but also on the level of oxidative stress.

### 2.4. Mitochondrial S-Glutathionylation and Circadian Oscillations

S-glutathionylation mediated by a low GSH:GSSG ratio appears to be overrepresented in mitochondrial proteins compared to the whole-cell proteome. Lind et al. [51] identified 43 protein substrates in the postmitochondrial fraction of immortalized ECV304 endothelial-like cells exhibiting cellular S-glutathionylation during diamide-induced oxidative stress. The substrates include protein chaperones and cytoskeletal proteins. The mitochondrial genome codes only 13 protein components. The 1119 nuclear-encoded genes currently identified as localizing in mitochondria correspond to only 6% of all protein-coding genes according to the Human Protein Atlas database (www.proteinatlas.org (accessed on 25 February 2023)), yet almost 30% of all altered cysteine sites displaying S-glutathionylation modifications were located in or associated with skeletal muscle mitochondria of young/aged mice [52]. S-glutathionylation temporarily inhibits mitochondrial ROS production and most metabolic pathways. including glycolysis, the tricarboxylic acid (TCA) cycle, fatty acid and amino acid oxidation and oxidative phosphorylation [53].

Under normal coupling conditions, CI, less so CIII, and CII to an even lesser extent, are considered the main sources of ETC ROS (Table 1). Even during oxidative eustress, not only are ROS generated by leakage of the respiratory ETC, but also non-ETC ROS are produced by mitochondrial enzymes of major metabolic pathways [53]. The use of isolated mitochondria from CI-deficient mice compared with wild-type controls demonstrated that superoxide/H_2_O_2_ rates from CI, depending on the substrate, can be far less than mitochondrial non-ETC ROS generated in mitochondrial multienzyme complexes [54]. Three non-ETC, high ROS emitters, of the many non-ETC sources of ROS [21], were the α-ketoglutarate dehydrogenase or oxoglutarate dehydrogenase complex (α-KGDH or OGDC, twofold higher than CI ROS), pyruvate dehydrogenase (PDH, fourfold CI ROS), and branched-chain keto acid dehydrogenase (BCKDH, eightfold CI ROS) [54]. Note that due to the specialized experimental assessments of these non-ETC sources of ROS, non-ETC ROS may be significant yet less than ETC sources in vivo. Like ETC ROS emitters CI-III, non-ETC ROS emitters, α-KGDH and PDH are targets for GSH modification, but not BCKDH [55].

These dehydrogenases are rate-limiting steps in mitochondria, as α-KGDH is a TCA cycle control point enzyme, affecting oxidative phosphorylation/ATP production, PDH connects glycolysis to the TCA cycle, and BCKDH catalyzes the catabolism of branched-chain amino acids, which is required for cellular protein synthesis. All three of these dehydrogenase activities are subject to roughly circadian changes, as observed in isolated mitochondria. The peak time of α-KGDH and PDH mouse liver mitochondria is in the early light phase (zeitgebers—ZT3 and ZT4, respectively) in mouse liver mitochondria [32]. Similarly, BCKDH approximately peaks at the end of the dark phase in hepatic mitochondria derived from rats that were previously fed during hours of darkness [56]. Maximum ATP production and carbohydrate oxidation are during wakefulness in humans (Section 3.4). Similarly, the peak activity of whole-body ATP consumption required for nucleolus enlargement, ribosome biogenesis and protein synthesis occurs in parallel with high oxygen consumption and after feeding during the wake phase [57,58]. Three points can be made with respect to these three dehydrogenases: first, they are significant non-ETC sources of ROS; second, two of the three dehydrogenases that are the highest ROS sources are redox-modified; and third, the activity and ROS production of all three dehydrogenases undergo circadian oscillations.

Redox signaling regarding glutathionylation can be complex. A decrease in the redox couple GSH:GSSG (the electron acceptor, the oxidized form of GSH, is glutathione disulfide, GSSG) for the most part results in protein S-glutathionylation, but this is not the case for UCP2/3. H_2_O_2_ induces glutathionylation of UCP2/3 and, surprisingly, GSH:GSSG in a more reduced state also promotes deglutathionylation of UCP2/3, hence UCP2/3 activation (Section 3.2) [59]. This activates proton leaks through UCP2/3 resulting in the dampening of mitochondrial H_2_O_2_ production. This mechanism has been suggested to serve as a “safety valve” to diminish H_2_O_2_ production in response to its higher-than-normal generation. The GSH removed from UCP2/3 was also suggested to play a role in restoring GSH:GSSG redox tone [59]. Together, this unique regulation of UCP2/3 by H_2_O_2_ and glutathionylation plays a part in maintaining overall cellular redox buffering. 

Protein S-glutathionylation results from the covalent addition of glutathione to a cysteine thiol, resulting in a net increase in molecular mass and negative charge. Like other posttranslational modifications, this results in changes in overall protein structure, activating or deactivating these proteins. Glutathionylation can deactivate proteins by blocking catalytic thiols but in other cases it can lead to activation. Some proteins also display unique regulatory attributes like the UCPs (as described above). Additionally, some of these proteins are activated/deactivated, respectively, by glutathionylation/deglutathionylation in response to different physiological states like light/dark cycles. For example, ANT, HIF-1α, and CII (a mitochondrial ETC enzyme connected to the TCA cycle) are protein S-glutathionylated or “PSSG-activated” exhibiting circadian rhythmicity (Table 1).

It has been shown that carbon-monoxide-generated ROS produce GSSG in isolated mitochondria, which can trigger the first PSSG-activated example—ANT glutathionylation. Consequently, ROS-enhanced GSSG levels facilitate ADP/ATP translocation by ANT; glutathionylated ANT also inhibits mitochondrial permeability transition pore activity and impedes the cell death process [60]. The second PSSG-activated example of glutathionylation (and phosphorylation), HIF-1α, occurs during hypoxia, facilitating cell survival [61]. HIF-1α both regulates and is regulated by circadian rhythmicity [62]. The third PSSG-activated example, CII, can independently provide reserve respiratory capacity, enhancing cell survival during steroidogenesis and hypoxia (see sleep apnea, Section 5.4.2) [63]. CII, along with CIII and CIV, regulates steroid metabolic processes involving mitochondrial proteins [64]. In addition, CII operates with the circadian-dependent TCA cycle [32], so greater CII activity may help explain the elevation of mitochondrial fatty-acid β-oxidation and TCA intermediates (e.g., succinate) during ultradian-cycling REM sleep, as observed in humans. This limits ATP production and thermogenesis (Section 5.4.6) [65,66]. Succinate-derived ATP creates less ROS than pyruvate-derived ATP. Higher-frequency REM-NREM cycling associated with species of smaller mass [67] possibly provides greater protection and recovery via CII, due in part to bypassing CI. Consequently, these rare PSSG-activated examples, ANT, HIF-1α and CII, are circadian oscillators that regulate the mitochondrial redox state and consequently are mitoprotective in nature.

A key question is: When during the sleep–wake cycle does this lead to ROS-regulated glutathionylation and the general dampening down of these enzymatic processes in humans? Pertinent to the circadian timing is the finding that in an ex vivo SCN-bearing rat hypothalamus, protein glutathionylation increased during the day, peaked in the evening, and had diminishing activity during the night [68]. A supportive parameter is the redox couple, GSH:GSSG having a low value when stressors and glutathionylation are high. Glutathionylated hemoglobin was 46% higher in hemodialysis patients than healthy controls [69]. Although it cannot be stated definitively, it appears from published animal research that ROS production and protein glutathionylation levels are highest during the high metabolic rate associated with wakefulness, and when redox parameters such as GSH:GSSG have lower values (Section 2.5).

### 2.5. Redox Couples and Circadian Oscillations

Several key redox couples demonstrate circadian oscillations, including reduced/oxidized forms, GSH:GSSG, cysteine:cystine, NADH:NAD^+^ coenzymes of nicotinamide adenine dinucleotide and NADPH:NADP^+^ coenzymes of nicotinamide adenine dinucleotide phosphate [70]. An unbalanced redox state with reductive stress can, like oxidative stress, damage proteins and lipids and form diffusible sulfur-centered radicals. The balance of the redox state depends on ROS generation and the production of reducing agents such as GSH, NADH, or NADPH. The high cellular ratios of GSH:GSSG (>100:1) and NADPH:NADP^+^ (~100:1) lead to a greater ability than NADH:NAD^+^ to reduce organic molecules. NADH:NAD^+^ has a ratio of about 1:10 in mitochondria and as low as 1:1000 in cytoplasm that leads to oxidative stress [71].

The redox couples GSH:GSSG and cysteine:cystine in adults have diurnal and meal-associated variations [24]. The human plasma GSH:GSSG redox states undergo weak circadian oscillations and this redox couple ratio is at its lowest, and hence most oxidized, in the early afternoon. The cellular GSH:GSSG redox couple can be >100:1, and is therefore mostly in its thiol-reduced form, dropping as low as 1:1 during oxidative stress in isolated mitochondria [72]. Production of the most abundant thiol GSH involves a two-step enzymatic process requiring ATP and cysteine. The cysteine:cystine ratio in human plasma peaks in the evening, drops during the night and increases 2–3 h after meals [24]. Hence, the trend in plasma is that the minimum GSH value and greatest oxidation of the GSH:GSSG redox states occurs around 13:30 in humans, seven hours after the cysteine minimum. The GSH:GSSG ratio was at its highest and most reduced in the early waking hours (around 8:00) and in the evening (17:30–21:30).

GSSG is reduced to GSH by GSH reductase facilitated by electron-donor NADPH or alternatively deglutathionylation involves enzymes such as GRX, TRX and SRX [37]. The cytoplasmic pentose phosphate pathway is important for redox homeostasis by providing NADPH biosynthesis. In response to ROS-mediated inhibition of glycolysis, some cell types divert glycolytic flux from the reversible, non-oxidation branch of the pentose phosphate pathway, which does not produce NADPH, into the oxidative branch of this pathway to produce NADPH from NADP^+^ [73]. Hence, the NADPH-facilitated reduction of the GSH antioxidant pathway is an important oscillatory, transcriptional, and translational regulator, governed to some extent by NADPH availability peaking in the circadian phases when animal species are resting, rather than active [74,75]. In summary, redox couples GSH:GSSG and NADPH:NADP^+^ (but not cysteine:cystine) accumulate lower ratio values, and hence are more oxidized during the active phase with restitution of higher ratio values during the resting phase.

## 3. Mitochondrial Energetics, Cellular Metabolism, and Circadian Rhythms

### 3.1. Introduction

Balancing ATP production against ROS generation is critical, especially considering that the ATP synthesized by mitochondrial CV rises linearly with the membrane potential; however, the ROS generated by the ETC rises exponentially with the proton gradient at a strongly polarized mitochondrial membrane potential [97,98,99]. This section describes the main types of anion carriers and anion carrier mediators that not only control the mitochondrial proton gradient but also regulate metabolic switching during sleep–wake cycling. The mitochondrial anion carriers, UCPs, regulate redox signaling by moderating antioxidant protection and by constraining mitochondrial superoxide and H_2_O_2_ formation [27]. The anion carriers are molecular mediators such as fatty acids and melatonin that regulate the circadian proton transport of UCPs. Also described in this section are circadian rhythms of mitochondrial respiration and metabolic cycling in which mitochondrial dynamics and cellular bioenergetics have a central role [100].

### 3.2. Anion Carriers

Mitochondrial coupling efficiency is reduced by a small inducible protein leak and a larger basal proton leak that is about 20–50% of the basal respiratory process, depending on the species and cell type [28,92,101]. Proton leaks are mainly attributable to the UCP and ANT subfamilies. ANT’s intrinsic uncoupling activity contributes up to two-thirds of the basal proton conduction in murine muscle mitochondria, independently of its exchange of ATP for ADP or fatty-acid-dependent proton-leak functions [102]. Similarly, UCP1–3 can be induced by anion carrier mediators, such as the thyroid hormone triiodothyronine (T_3_) and long-chain fatty acids [103,104]. Notwithstanding, the focus will be on the UCP subfamily of the mitochondrial anion carrier protein family.

The five mammalian members of the mitochondrial UCP family have different concentrations and roles in various tissues and cell types. UCP1 in brown adipose tissue causes a mitochondrial “proton leak” that is virtually exclusive to heat production. UCP1 has a greater thermogenic role in infancy than in adulthood [105]. UCP2–5 primarily create low ROS conditions, yet also contribute to body heat and stimulate adaptive thermogenesis on cold exposure and high-fat diets [106]. UCP2/3 are highly expressed in adipose tissue and skeletal muscle, which are important sites of both thermogenesis and substrate oxidation [107]. UCP2 dissipates the mitochondrial proton gradient in the brain, liver, and other organs by transporting protons across the inner mitochondrial membrane [108]. In the process, UCP2 lowers membrane potential, ROS formation, and inflammation. UCP2/4/5 are found in neurons, but not UCP1 or UCP3 [109,110]. The brain is an organ with a very high energy demand, normally relying on aerobic glucose-fueled energy metabolism. Considering the necessity of the high metabolic rate of the brain and the need to protect postmitotic neurons and their mitochondria from excess ROS, it is not surprising that the brain has specialized uncoupling mediators.

Although UCPs are acknowledged as subtle regulators of redox signaling [27], there is a debate as to whether UCPs have greater influence as a metabolic switch and a lesser influence on uncoupling [111]. UCP2 activity promotes mitochondrial fatty-acid β-oxidation, yet limits mitochondrial catabolism of glucose-derived pyruvate, which is a major energy source of proliferative cells [112]. Circadian-like UCP2 activity linked to glucose-induced ATP production has been observed in mouse pancreatic islets and β cells, with the upregulation of UCP2 in the resting (light) phase preventing hypoglycemia [95]. Whatever the significance of UCPs’ role as a metabolic switch, there are many indirect indications that anion carrier activity is generally greatest, with exceptions, during the sleep phase, such as fatty-acid β-oxidation dominance during this time and the action of anion carrier mediators, as shall be described.

### 3.3. Anion Carrier Mediators

The proton conductions of certain anion carriers such as UCPs have been shown to be regulated by numerous anion carrier mediators such as ROS, GSH, fatty acids, S-glutathionylation, thyroid hormones, and melatonin. Superoxide and lipid peroxidation products share a common pathway activating UCPs to increase uncoupling by proton conductance and by transmembrane anion transport of protonated fatty acids [113,114]. It has been shown that superoxide-mediated activation of UCP1, -2, or -3 requires fatty acids [113]. The proton conductance of UCP1/2/3 is inhibited by purine nucleotides and enhanced by long-chain fatty acids (and nutrient availability [95]), polyunsaturated fatty acids being more effective than saturated fatty acids [115]. In human plasma, the concentrations of triacylglycerols and diacylglycerols rose during sleep, peaking near wake time [116]. As detailed in Section 2.4, UCP2/3 are inactivated by S-glutathionylation, and a rise in H_2_O_2_ uniquely results in removal of the glutathionyl moiety to activate the proteins [59]. UCP2/3 have exposed thiols, like a substantial fraction of the mitochondrial proteome, and can be covalently modified by GSH [92,93]. Therefore, UCP2/3 mediated leak respiration is uniquely positioned to undergo activation in response to diurnal fluctuations in redox tone, being more active and deglutathionylated at night in comparison to the day for the prevention of oxidative stress. However, as noted here, UCPs are also positively regulated by fatty acids, which also increase in the sleep cycle. Together, the increased availability of fatty acids and H_2_O_2_ [10,116], render UCPs more active during sleep than wakefulness. 

**Figure 2:**
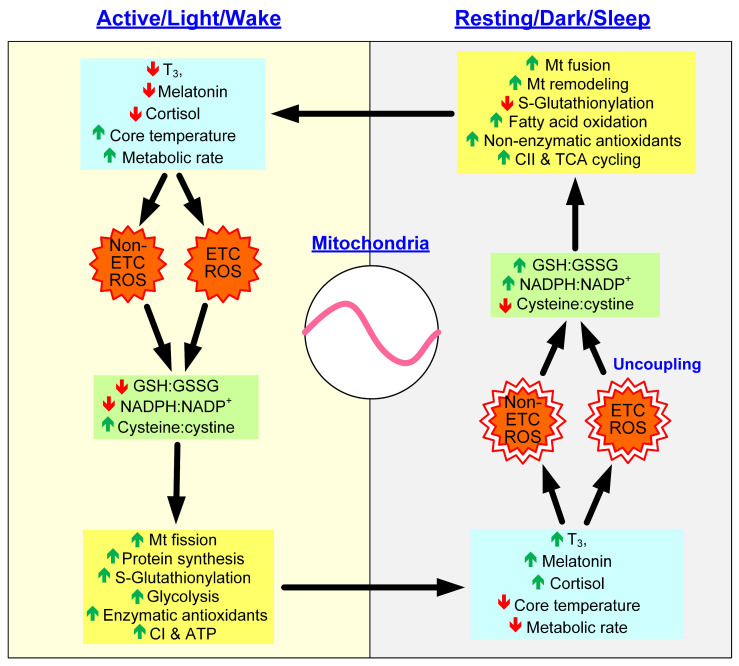
Proposed sleep–wake interaction of reactive oxygen species (ROS), redoxome, anion carrier mediators, uncoupling and temperature related to cellular processes of endothermic animals in general, but especially related to mitochondrial (Mt) function and the electron transport chain (ETC). Process activity is shown raised 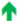 or lowered 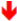 for parameters such as glutathione (GSH), the oxidized form of GSH (GSSG), metabolic pathways, mitochondrial complexes I and II (CI & CII), NADPH:NADP^+^ agents of nicotinamide adenine dinucleotide phosphate, tricarboxylic acid (TCA) cycle and triiodothyronine (T_3_).

The hypothalamic–pituitary–thyroid axis, which is circadian and regulated by the SCN central pacemaker, is a prominent mediator of the proton leak [117]. The focus here is on thyroid hormones, as they have important roles in basal thermogenesis, metabolism, cell cycle and growth, but also constitute a major regulator of the protein leak, in which UCPs play an important although debatable role [105,118,119,120]. In concordance with these activities, thyroid hormones in some measure regulate the circadian-dependent resting metabolic rate, and hence energy levels and core temperature [121]. This is in part due to thyroid hormones directly affecting mitochondrial biogenesis, oxygen consumption and oxidative phosphorylation. The thyroid hormones produce 100% of thyroxine (T_4_) and ~20% of T_3_. T_4_ is a precursor to T_3_, with ~80% of T_3_ generated from T_4_ in non-thyroidal organs. The pituitary thyroid hormone, or thyroid-stimulating hormone (TSH), stimulates T_4_ production. Thyroid hormones TSH, T_4_, and T_3_ exhibit circadian rhythms [122]. Free T_3_, similar to cortisol macrovariability, stays above the rhythm-adjusted mean or mesor from 22:00 to 10:00, with its zenith in the early morning (~4:00) (Figure 2). Therefore, thyroid hormones have a major effect on the integration of circadian-modulated thermoregulation and mitochondrial function, including uncoupling of oxidative phosphorylation.

Melatonin, an anion carrier mediator, is a pineal gland hormone having many functions, including regulation of mitochondrial dynamics. It is better known for its role in sleep regulation [123]. Melatonin is lesser known for protecting against ETC ROS by acting on UCPs to dissipate the protein gradient and as an antioxidant scavenging ROS/RNS [124]. Melatonin is excreted in a diurnal manner, and reciprocally regulates clock proteins in the SCN and peripheral tissues. Melatonin is excreted mainly in darkness and is at peak levels in the middle of the night. Melatonin is mainly synthesized in mitochondria, with this organelle supplying the required cofactor/substrate, acetyl-CoA. As demonstrated in pinealocytes, brain neurons and other cell types, melatonin regulates mitochondrial dynamics [124]. Mitochondria have a half-life in the order of days, involving fission and fusion [86]. Melatonin inhibits fission via DRP1. DRP1 is recruited from the cytosol to the mitochondrial outer membrane [87]. DRP1-mediated fission, characteristic of impaired mitochondrial function and increased ROS production, leads to a fragmented mitochondrial network and mitophagy. Therefore, the lower melatonin levels during daytime allow fission to take place during wakefulness [100].

Mitochondrial fusion, which is characteristic of improved mitochondrial function and efficient ATP production leading to mitochondria forming tubular networks, accompanies the melatonin secretory peak [124]. Fusion, the dynamic state most beneficial to mitochondria, likely occurs preferentially during sleep and fasting (Figure 2). In addition, melatonin in a circadian and dose-dependent manner, exerts a mitoprotective effect by supporting the ETC flux and preventing a breakdown of the mitochondrial membrane potential when compromised by a calcium or H_2_O_2_ overload [123,124]. In summary, melatonin especially protects mitochondria during the sleep phase by its antioxidant properties, optimization of membrane potential, and promoting fusion.

Two other uncoupling mediators are cortisol and sterols. Glucocorticoids have been shown to activate UCP2 function in hyperglycemic, microvascular endothelial cells and thus inhibiting glucose-induced mitochondrial ROS [125]. The glucocorticoid steroid stress hormone, cortisol, peaks in the early morning in diurnal animals and humans upon awakening and declines during the day in a circadian manner [126]. Sterols in animals, including cholesterol, also have a circadian-related role in lowering mitochondrial oxygen consumption and may protect against extracellular oxidant attacks penetrating the plasma membrane [127,128]. In addition, UCP1–3 are also regulated by retinoids, catecholamines and rexinoids [107]. Consequently, GSH, fatty-acid anions, thyroid hormones, melatonin, cortisol, and sterols are known to modify (likely in a circadian manner) uncoupling and the proton leak.

### 3.4. Cellular and Mitochondrial Metabolic Circadian Rhythms—Futile Cycle Avoidance

Examination of respiratory parameters can provide proxy quantification of the basal metabolic rate during sleep–wake phases. The ratio of the expired volume of carbon dioxide to the volume of consumed oxygen, known as the respiratory quotient (RQ), is ~0.7 for lipid metabolism and unity for carbohydrates. Fatty-acid oxidation occurs in several cellular sites (chiefly occurs in mitochondria) and provides nearly twice the energy per gram of carbohydrate oxidation. Circadian variation in metabolism and physiology involves regulation of, and regulation by peroxisome proliferator-activated receptors (PPARs). For example, PPARα promotes hepatic fatty-acid β-oxidation and ketogenesis in response to fasting [116]. Increased long-chain acylcarnitines in human breath during the sleep (fasting) phase indicates a greater reliance on fatty-acid β-oxidation than during sleep, aligning with RQ studies [65]. The resting energy expenditure RQ varies with circadian phase in humans: RQ is at its zenith in the biological morning, being ~25% greater than at its nadir in the late evening [9]. This corresponds in normal human subjects to glucose utilization being at its highest during the wakeful state (when blood glucose levels are lowest) and at its lowest utilization during sleep [129]. Greater reliance on fatty-acid β-oxidation in mitochondria while sleeping provides a more efficient form of ATP production than carbohydrate oxidation; however, this may be at the cost of increasing oxygen toxicity, necessitating greater mitoprotective uncoupling, as explored in Section 5.2 and Section 6.3.4.

The glucose–fatty acid or Randle cycle undergoes reciprocal interactions between glucose and lipid oxidation and has an adaptive thermogenic role, especially in skeletal muscle [130]. Two substrate pathways that can run concurrently in opposite directions dissipating energy in the form of heat are called substrate or futile cycles. The cellular Randle cycle and mitochondrial cycle, comprising fatty-acid β-oxidation and mitochondrial fatty-acid synthesis (mtFAS; note FAS also occurs in the cytoplasm), are both considered futile cycles [130,131]. Experiments in mouse liver demonstrate strong temporal and spatial organization of mitochondrial and nuclear lipids with diurnal, coregulated and opposite rhythmic phases [132]. We speculate that both the Randle cycle and mitochondrial fatty-acid β-oxidation/mtFAS cycling undergo, at least in part, circadian-related reciprocal regulation.

The glucose–fatty acid cycle in muscle and adipose tissue, proposed by Randle and colleagues in 1963 [133], separates in a temporal fashion the potential competition between substrates for mitochondrial oxidation [130]. Based on RQ and other experimental observations in humans, glucose oxidation and fatty-acid oxidation are principally activated during the active and resting phases, respectively. Fatty-acid β-oxidation is essential in providing restitution of the major portions of the immune system [134]. The human innate immune system produces higher levels of circulating naïve and memory T cells and proinflammatory cytokines during sleep [135]. These T cells require oxidative phosphorylation and fatty-acid oxidation, which are most active during the sleep period [136]. Futile cycles generate either heat or ATP as in the proposed substrate cycle of de novo cytoplasmic FAS and concurrent fatty-acid oxidation in memory T cells [137]. Therefore, temporal separation of substate cycles should be considered a possibility in view of the differential mitochondrial–nuclear metabolic activities of the immune system during sleep–wake cycling.

Similarly, the interacting substrate pathways of mitochondrial fatty-acid β-oxidation and mtFAS comprise a potential cycle of temporal separation. Mitochondrial acetyl-CoA, produced by fatty-acid β-oxidation, is a substrate of both the mtFAS pathway and the TCA cycle, the latter leading to ATP production [131,138]. The medium-chain fatty-acid octanoic acid/caprylic acid (precursor of α-lipoic acid) is the sole product of the mtFAS pathway. Antioxidant α-lipoic acid is potent in both its reduced and oxidized forms, and is also an essential cofactor of mitochondrial enzymes α-KGDH, BCKDH, and PDH (Table 1). MtFAS not only regulates FeS cluster synthesis, mitochondrial translation, and ETC and supercomplex assembly [138] but also regenerates nonenzymatic antioxidants such as vitamin C, vitamin E, and GSH [139]. MtFAS, a significant coordinator of mitochondrial oxidative metabolism [138], is redox-regulated by both S-nitrosylation and S-glutathionylation [48,140]. These cysteine oxoforms have a high degree of circadian oscillation (Section 2.2 and Section 2.4). Consequently, we speculate that the mitochondrial fatty-acid β-oxidation and mtFAS substrate pathways are an example of a potential futile cycle that undergoes temporal separation, and consequently are critical components of mitochondrial differential activities during the sleep–wake cycle, or even sleep phases/states (Section 5.4.6).

## 4. Core Temperature, Heat Stress, Mitochondrial Oxidative Stress, and Circadian Rhythms

### 4.1. Introduction

The core temperature in humans drops to its lowest during early morning sleep, 36.5 °C at 4:00, and rises to its highest when awake in the evening—37.4 °C at 20:00 [14]. The optimal temperature of human cells lies within a small temperature range. Mitochondrial ETC activity has an optimal temperature of around 50 °C. Mitochondria are physiologically maintained at least 6–10 °C above core body temperature when the ETC flow is high, providing an estimated 60% of NADH oxidized as heat and 40% as ATP when ATP synthesis is maximal [141]. Mitochondria are exquisitely tuned to high performance, especially those in neurons. As shown in this section, this renders mitochondria, like stem cells, extremely vulnerable to high- and low-temperature stress and the need for the microenvironmental conditions of mitochondria to be just right [142]. As with oxygen levels that are either too hypoxic or too hyperoxic, conditions that generate excessive oxidative stress [22], a temperature outside a certain physiological range (demonstrated in an ectotherm, depending on cellular requirements) also produces excessive oxidative stress, especially due to ROS from CI [143].

### 4.2. Heat Shock Response

The heat shock response protects cells and their organelles from stressors such as high temperature, infections, and oxidative stress. This response activates molecular chaperones, namely heat shock proteins (HSPs), that prevent or reverse protein unfolding/misfolding and enable protein repair, protein resynthesis and nuclear DNA damage repair [144]. Note, S-glutathionylation is a regulator of the unfolded protein response [49]. A heat shock response is enacted by chaperones, preventing protein unfolding/misfolding, which include the HSP60 and HSP70 family that undergo reversible cysteine modifications, including glutathionylation [47,49]. The redox state has a major influence in activating the heat shock response to cope with elevated temperatures, H_2_O_2_, acidic-pH, ionizing radiation, and other proteotoxic distresses. Stress and GSH when oxidized cause the trimerization of heat shock factor 1 (HSF1) a key step in HSF1 activation [145]. However, under normal diurnal stresses, HSF1 is the main regulator of the circadian transcription of heat shock genes, and reciprocally interacts with the HSP90 and HSP70 families found throughout cells. The nuclear HSF1 level in non-hibernating chipmunks, a diurnal mammal, increases when their body temperature is rising in the active phase (HSF1 is also activated during the wake and arousal phases of the hibernation season); and HSF1 decreases when body temperature falls during the resting phase of the sleep-wake cycle [146]. These examples show that circadian redox and temperature regulation is an integral part of the heat shock response.

### 4.3. Mitochondrial Temperature and ROS Dependence

Heat stress, as demonstrated in *Drosophila melanogaster*, activates reverse-direction transport of ETC flow, causing CI to generate excess ROS [143]. Studies involving the immersion of male humans, mean age 20, at three different water temperatures, 25 °C, 39 °C, and 42 °C for 10 min, and similarly in animal experiments, demonstrate that thermal stress affects the blood GSH:GSSG ratio [147]. Heat stress causes CI dysfunction, less ATP synthesis, more oxygen consumption, and greater ROS production [148]. Control of heat stress is essential for limiting oxidative stress for efficacious mitochondrial function.

Somewhat counterintuitively, it appears from the scientific literature that the lowering of core temperature during sleep (and during hibernation, Section 5.5) also increases oxidative stress in mitochondria. Lower temperatures of incubated mitochondria significantly increase oxidative stress as quantified by the production of lipid peroxidation products [149]. When isolated murine brain mitochondria were subjected to a fall in temperature from 37 °C to 35 °C (compared to a ~1 °C human sleep–wake temperature change), the oxygen consumption rates of phosphorylating respiration phase (maximal rate of oxygen consumption, state 3) and resting respiration phase (leak state 4) decreased by 29% and 21%, respectively, for this 2 °C drop in temperature [15]. Surprisingly, lowering the temperature by 2 °C also increased O_2_^•−^ and H_2_O_2_ production in respiratory states 4 and 3 by 62% and 98%, respectively, and increased hyperpolarization of mitochondrial membrane potential by about 2% and 16%, respectively, though at statistically insignificant levels. A greater 5 °C drop in temperature to 32 °C caused significant hyperpolarization of the mitochondrial membrane potential in both respiratory states 4 and 3 in isolated brain mitochondria. Similarly, when the temperature was lowered by 15 °C in both porcine perfused kidneys and isolated kidney mitochondria, oxygen consumption decreased by ~57%, whereas the mitochondrial ROS production was reduced by only ~16% [149].

This trend of higher ROS production being linked to a lower mitochondrial temperature may be more characteristic of organs of high basal metabolic activity per unit mass, such as the brain and liver [150]. A temperature change from euthermia to hypothermia (from 35 °C to 25 °C) increased H_2_O_2_ production in isolated mitochondria from rat skeletal muscle (tissue of lower metabolic activity) in a nonphosphorylating state (state 4); however, no significant changes in H_2_O_2_ were observed for phosphorylating mitochondria (state 3). It is of note that this section refers to studies involving isolated mitochondria, yet this medium omits the effect of extramitochondrial thermoregulators such as thyroid hormones and melatonin. Notwithstanding, the experiments described showed that the lowering of mitochondrial temperature resulted in a far greater percentage increase in ROS production compared to the percentage loss of mitochondrial respiration, especially for organs of high basal metabolic activity.

### 4.4. Redox/Temperature-Dependent Peripheral Clocks

Peroxiredoxins are antioxidant proteins and enable peripheral clocks that are both redox- and temperature-regulated. PRXs reduce intracellular peroxide and peroxynitrite levels, especially H_2_O_2_, by a cysteine residue being oxidized to sulfenic acid, which then reacts with another cysteine residue to form a disulfide bond that is reduced by TRX [74]. PRXII is abundant in anuclear red blood cells and PRXII can be hyperoxidized (PRXII-SO_2/3_) and inactivated when eliminating H_2_O_2_. O’Neill and Reddy [74] reported that hyperoxidized PRXII undergoes redox circadian entrainment in human red blood cells. Similar PRX rhythms were seen in mouse nucleated fibroblasts. PRX-SO_2/3_ levels were at high levels at low temperatures, and at low levels at high temperatures, which may have implications regarding the effect on PRX levels by circadian body temperature cycles.

There is considerable evidence to support an important role of the mitochondrial enzyme PRXIII in cellular timekeeping by peripheral clocks (Section 1) [16,90]. PRXIII undergoes reversible inactivation via hyperoxidation and is reduced by SRX, which has a disulfide link to HSP90. To help limit ROS, especially H_2_O_2_, PRXIII and SRX are regulated by both S-sulfinylation and steroidogenesis: these two processes like PRXIII and SRX also undergo circadian rhythms [16,90]. The central and peripheral clocks do not act independently: there is evidence of a reciprocal relationship between central and peripheral oscillators involving cellular spatiotemporal variations of the redox code [36,151], as well as thermal regulation [152].

## 5. Implications of the Hypothesis

### 5.1. Introduction

It is important to note that the normal reversibility of S-glutathionylation and S-nitrosylation is contingent upon the suppression of oxidative and nitrosative distress. This requirement is at risk with perturbed sleep–wake cycling associated with advanced aging and certain diseases, which have deleterious effects on circadian redox rhythms. The redox–bioenergetics–temperature and differential mitochondrial–nuclear regulatory hypothesis has several implications by providing further insight on uncoupling, avoidance of metabolic futile cycling, sudden infant death syndrome (SIDS), and old age and some age-related diseases. In addition, the hypothesis provides added understanding of torpor and hibernation in animals that share some similarities with circadian sleep–wake cycles. Lastly, we show that the hazards of spaceflight align with the perturbation of tripartite-interactome signaling. For each example, the three interactive components of redox–bioenergetics–temperature cycling will be addressed, followed by evidence for perturbed circadian rhythms.

### 5.2. Reasons for Uncoupling

Various hypotheses have been proposed to explain the reasons for respiratory uncoupling and proton leak. Here, three hypotheses will be considered and discussed. First, the “mild uncoupling” hypothesis is centered on a small reduction in proton motive force having a marked effect in dampening exponentially dependent superoxide formation [97,98]. Second, Brand [153] proposed, contrary to others at the time, that thermogenesis is not the primary function of the proton leak. Brand’s hypothesis of “uncoupling to survive” suggests that the main effect of the “mitochondrial futile proton cycle” is not only thermogenic but related to the mediation of free radicals, though with the sequitur that this process increases life span. There is evidence to support smaller mammalian species having not only a greater metabolic rate but also more proton leakage to counter increased mitochondrial H_2_O_2_ production [153,154,155]. A third hypothesis, “redox-optimized ROS balance” by Cortassa et al. [156], is based on the minimizing of mitochondrial H_2_O_2_ when energetic performance is high, as indicated by respiratory oxygen consumption and ATP synthesis.

These three extant uncoupling hypotheses do not account for circadian variations in oxygen consumption or metabolic rate, and likely parallel changes in proton and electron leaks. However, Skulachev [97], in support of the “mild uncoupling” hypothesis, proposed that anion carrier mediators provide respiratory protection resulting from the lowering of the intracellular oxygen concentration that is especially relevant in the resting state (no mention of circadian rhythm or the sleep–wake states) when oxygen consumption is low. We make the case that another major role for uncoupling is as an integral part of the circadian redox–bioenergetics–temperature regulation of the sleep–wake cycle because anion carriers such as UCPs in mammals are regulated by anion carrier mediators such as ROS, fatty acids, S-glutathionylation, thyroid hormones and melatonin (Section 3.3, Figure 2). In general, as UCPs are preferentially activated by anion carrier mediators during sleep, one can speculate that UCPs participate in the temporal separation of substrate cycles. Acknowledging that UCPs promote fatty-acid β-oxidation yet limit carbohydrate oxidation [112] may have implications for UCP involvement in the temporal separation of the Randle cycle and cycling of fatty-acid β-oxidation and mtFAS (Section 3.4). This time-managed control of uncoupling helps facilitate sleep–wake redox–bioenergetics–temperature cycling and likely temporally separates potential futile cycling of the metabolism. Consequently, UCPs may have a major role in providing the differential and restorative physiological characteristics of the sleep–wake phases.

### 5.3. Sudden Infant Death Syndrome and Impaired Circadian Rhythm Development

The neonatal mortality rate in general is far higher than death rates at later stages of human development, and besides dominant socioeconomic factors, immature or perturbed redox–bioenergetics–temperature cycling may play a role. During a normal pregnancy, the fetus develops in a relatively hypoxic environment and slightly elevated maternal core temperature. At birth the newborn is first exposed to normoxia and cold extrauterine temperatures and consequently subjected to accelerated oxidative stress, to which the brain is particularly vulnerable [157]. Levels of ROS in human leukocytes and total antioxidants in plasma are highest in newborns [10], when their circadian rhythm is not independently entrained from that of their mothers, and their circadian rhythm does not develop until 3 to 6 months of age. During the period of highest neonatal mortality, 1–2 days postpartum, neonates have a relatively static core temperature of about 37 °C [158]. The acquisition of circadian entrainment and sleep–wake core temperature and redox cycling early in infancy may help constrain excessive ROS later in life, when ROS in leukocytes has been observed to be at lower levels and varying little between 20 and 80 years old [10]. Immature redox–bioenergetics–temperature cycling does not provide the protective and restorative benefits of the differential activities that are derived by an individual possessing circadian rhythms. It is proposed that one advantage of this lack of circadian rhythmicity is the parallel lack of time separation of the Randle and other substrate cycles in the skeletal muscle of newborns [130], thereby providing much-needed heat by futile cycling.

Anion carriers are active during all developmental stages to varying degrees. Anion carriers such as UCP1–3 are found in various tissues and at varying activity levels in the fetus, and in newborns, children, and adults [159,160]. Anion carrier mediators such as thyroid hormones and melatonin affect thermogenesis, core temperature and ROS levels. An infant’s wake–sleep cycling appears to develop in parallel with increasing circadian melatonin levels (newborns’ serum concentrations <1/10th that of 1–3 year old) [123] and oscillations in core temperature, metabolic rate and ROS [10]. Serum TSH levels peak within one day postpartum and fall several-fold within days. These maximal TSH levels would be expected to cause respiratory uncoupling and other thermoregulatory effects (Section 3.3). It has been demonstrated in calves that a daily rhythm of body temperature is lacking at birth and develops in the first two months of life [161]. Similarly, humans during the first three to six months of development develop lower nighttime core temperatures, sleep fewer hours daily and acquire a more regular sleep–wake cycle [123].

SIDS is multifactorial and sleep-related, with suggested causes including impaired arousal from sleep, brain abnormalities and hyperthermia [162]. There is evidence from animals and humans that newborns that do not adopt good circadian entrainment may be vulnerable to SIDS, and that long-term effects of poor circadian entrainment can be psychiatric disorders such as autism and schizophrenia [163]. Two prominent SIDS biomarkers have a direct participation in sleep–wake cycling and associated arousal. Low levels of butyrylcholinesterase (BChE), a measure of autonomic function, were measured in dried blood spots taken 2–3 days after birth [164]. Another SIDS biomarker, serotonin, is a monoamine neurotransmitter and SCN regulator [25] and is mostly produced by the gut enterochromaffin cells, but also by raphe nuclei in the brain stem. Postmortem levels of serotonin in infants diagnosed as a SIDS death, compared with autopsied controls, were at low levels in the brainstem and high levels in serum [165]. Another possible link to malfunctioning circadian rhythms causing sudden unexpected death in infants is that hereditary fatty-acid-oxidation disorders can lead to metabolic decompensation [166]. The circadian demands on fatty-acid oxidation presumably have a greater impact if malfunctioning during sleep when fasting. It is proposed that SIDS, as there is evidence of inadequate perinatal maturation of the circadian rhythm during this critical developmental period, may be better understood and treated by preventive circadian entrainment accounting for the interactions of centralized/peripheral circadian oscillators, core temperature, and the redoxome to minimize oxidative distress and maximize differential metabolic activities during an infant’s sleep–wake cycling.

### 5.4. Old Age and Associated Disease States

#### 5.4.1. Impaired Redox–Bioenergetics–Temperature Regulation

The robust daily amplitude and rhythmicity of redox–bioenergetics–temperature cycling in the young, become less pronounced or disturbed in old age, leading to compensatory changes in mitochondrial function, cellular metabolism, and cell survival associated with the adverse health effects of aging. Redox conditions are also less stable in older adults aged 18 to 86 years [24]. There is a 1.8-fold greater mean diurnal variation of the cysteine:cystine redox ratio in persons aged ≥60 years than that in persons aged ≤40 years. The GSH:GSSG ratio in human plasma is stable until about 45 years of age, then declines linearly [167]. The instability of redox couples may be partly due to the low-grade inflammation and oxidative stress associated with the immunosenescence of old age [168]. There are many reports showing the disturbance in respiratory uncoupling with aging, metabolic syndrome and neurodegenerative diseases. Thermoregulation is also impaired in the elderly due to many factors, including reduced sweating, slower metabolism, and thinner fat layer under the skin [169]. Yet another factor could be the lack of a strong amplitude of the circadian core body temperature, putting survival at risk, as seen in the neonate. Healthy older adults compared with younger ones have a core body temperature that is lower and has an oscillation amplitude that is also less, by a third [170,171]. Consequently, these observations on aging-dependent perturbed circadian rhythms and sleep–wake cycles implicate all components of the redox–bioenergetics–temperature cycling.

#### 5.4.2. Redox Cycling

Various age-related diseases, including metabolic syndrome, neurodegenerative diseases, and some cancers, compared to healthy aging, indicate dysfunction of the redox–bioenergetics–temperature regulatory components with variable weighting. Regarding abnormal redox cycling, the plasma GSH:GSSG ratio and redox potential are decreased in patients with obesity, hypertension and metabolic syndrome [172]. Diminished or abnormal GSH levels in blood and brain samples have been reported for five neurodegenerative disorders: amyotrophic lateral sclerosis, Alzheimer’s disease, Friedreich’s ataxia, Huntington’s disease, and Parkinson’s disease [77]. Metabolic syndrome and neurodegenerative diseases in both cases are characterized by excessive ROS/RNS. This oxidative distress perturbs redox regulation and thiol-disulfide homeostasis metabolic syndrome and neurodegenerative diseases, causing an irreversible oxidative modification of proteins, especially S-glutathionylation, which likely contributes to onset and progression [173,174].

Abnormally high levels of triglycerides in blood, the main constituent of body fat, have a profound effect in lowering skeletal muscle UCP2/3 activities, which is especially relevant in morbidly obese subjects [175]. Aging and age-related disorders cause perpetual ^•^NO production and S-nitrosylation of SIRT1. This nitrosative stress can induce chronic inflammation and apoptosis, contributing to sleep–wake disturbance [42,176]. For example, excess amyloid-β peptide in the brain of Alzheimer’s disease patients (who are prone to sleep disorders) promotes high levels of ^•^NO that lead to S-nitrosylation of the mitochondrial fission protein DRP1, causing mitochondrial fission/fragmentation, mitophagy and neuronal damage [44,85,177,178]. Therefore, reports on metabolic syndrome and neurodegenerative diseases indicate disturbed redox-regulated rhythmicity, one of the redox–bioenergetics–temperature regulatory components of sleep–wake phases.

Mild sleep-related hypoxemia disorders in humans is not uncommon and includes sleep apnea linked to oxygen desaturation events [179]. Obstructive sleep apnea in patients is characterized by repeated episodes of intermittent hypoxia resulting in impaired sleep arousal, systemic inflammation and elevated oxidative stress that raise the content ratio of the mitochondrial DNA (mtDNA) to nuclear DNA in blood cells [180]. Inflammation and hypoxia are interdependent, and there is a mutual intertwining of the hypoxia signaling pathway with redoxome cycling. For example, it has been shown in zebrafish fibroblasts that hypoxia (1% oxygen) perturbs the normal cycling of glycolysis and the major cellular redox systems, namely the circadian oscillations of cofactor pairs NADH:NAD^+^, NADPH:NADP^+^, and oxidized PRX levels [83].

#### 5.4.3. Bioenergetics/Respiratory Cycling

That UCPs are a critical component of the circadian interaction between oxidative stress and the redoxome is particularly indicated by diseases where circadian rhythm disruption and hypo/hyperglycemia play a role, such as metabolic syndrome [181]. Hyperglycemia, hypertriglyceridemia, hypertension, obesity, and low aerobic capacity are characteristics of metabolic syndrome, and are associated with heart disease, stroke, and type 2 diabetes [182]. As mentioned earlier, rhythmic UCP2 in pancreatic β cells prevents hypoglycemia [95]. Under normal circumstances, UCP2 can provide relief from oxidation stress of endothelial cells under hyperglycemia by redirecting glucose into macromolecule synthesis with greater utilization of the pyruvate oxidation and the TCA cycle [183,184].

Functioning mild respiratory uncoupling involving UCPs is an especially critical requirement for attenuating ROS in the heart and brain. However, too low a depolarization of mitochondria below a certain threshold, which may involve ANT, leads to mitopathy [185,186]. The loss of uncoupling was confirmed experimentally in the brains of mice with homologous UCP2 deletion (UCP2^−/−^) that showed increased ROS production, increased mitochondrial fission markers, and decreased fusion markers, principally affecting neurons rather than astrocytes [108]. The greater the electrochemical proton gradient in neuronal mitochondria the more ATP is synthesized. However, the downside is that calcium-induced hyperpolarization can cause exponentially-dependent production of mitochondrial superoxide [99]. These studies demonstrate that for optimal mitochondrial function, the electrochemical proton gradient is kept within a narrow range or otherwise leads to diseases of organs such as the heart and brain.

#### 5.4.4. Thermal Cycling

Old adults in general and patients with metabolic diseases and neurodegenerative disorders are more prone to poor thermoregulation and also a deficient heat stress response, including the ability of HSPs to improve glucose homeostasis or to reduce oxidative and proteotoxic stress [182,187]. Although different studies come to different conclusions, obese participants may have a higher mean body temperature than normal weight participants [170]. In addition, circadian thermoregulation of body temperature is also a problem for older adults in general, but particularly for patients with various forms of dementia and Parkinson’s disease [171]. The amplitude of circadian core body temperature is reduced in healthy older adults and several neurodegenerative diseases [188]. The amplitude of circadian core body temperature is reduced in patients with Alzheimer’s disease, yet their average core temperature is elevated by 0.1 °C. Emphasizing the importance of the benefits to the adult population of a lower core temperature achieved during adequate sleep, a lower body temperature may be a biomarker of longevity as observed in normal old age and calorie restriction studies [170].

#### 5.4.5. Circadian Rhythms

Sleep and circadian rhythm disruptions cause an earlier onset or greater severity of many chronic diseases, including metabolic syndrome, neurodegenerative diseases, and some cancers [177,189,190]. There is circadian rhythmic cross talk between intestinal microbiota, the metabolism, and the immune system that if chronically disrupted can elevate the mitochondrial levels of oxidative and metabolic stress, which can advance autoimmune disorders such as inflammatory bowel disease [191]. Gut microbiota dysbiosis can lead to damage-associated molecular patterns (DAMPs), inflammasome activation and excessive ^•^NO production that is immunosuppressive. ^•^NO-mediated S-nitrosylation of mitochondrial CI-V and DRP1 can lead to mitophagy (Section 5.4.2) [178]. The risk of developing metabolic syndrome and cancer is associated with night-shift workers such as nurses, likely due to circadian rhythm disruption [192]. Sleep disruption, systemic inflammation, metabolic changes and cachexia are common symptoms of cancer [193]. A prime function of the circadian rhythm is tumor suppression, for example via transcriptional factor, nuclear factor erythroid 2-related factor 2 (Nrf2) that controls antioxidant and anti-inflammatory responses [194]. It has been demonstrated, particularly in breast cancers, that tumor cells disrupt the normal circadian rhythm. Circadian dysregulation of the DNA response was shown in night-shift workers as opposed to the daytime enrichment in five of the six key DNA repair gene transcripts (peaked between 4:00 and 13:00) in the leukocytes of day-shift workers [195]. The role of nonmutational stages in carcinogenesis is often underestimated, as driver gene mutations make up only a third of the prediagnosis stages in US cancers [196]. Therefore, circadian rhythm disruption deserves serious consideration as a stage in advancing cancer development.

#### 5.4.6. Ultradian Rhythms

Although the focus is on circadian cycles, shorter physiological cycles (ultradian cycles) also participate in redox–bioenergetics–temperature regulatory processes. Ultradian cycles, superimposed on circadian ones, have been observed for plasma growth hormone (raised during sleep, encouraging muscle repair/growth), adrenocorticotropin hormone, cortisol, TSH, testosterone, and prolactin hormone [197]. The plasma glucose concentration and insulin secretion rate undergo ultradian oscillations of 80–130 min in males. The ultradian amplitudes and frequency of glucose, insulin, and plasma cortisol are very similar. Insulin resistance and hyperglycemia are linked to sleep loss, cause oxidative distress, and impair mitochondrial ATP production in the heart [198,199]. It has been demonstrated that excessive ROS activates UCP2, which impedes glucose-stimulated insulin secretion by glucose-sensing pancreatic β-cells [27,94]. Hence, the amplitude of the ultradian rhythmicity of glucose and insulin weakened by aging, obesity and sleep loss [129] leads to impaired mitochondrial function.

Ultradian rhythms are particularly important during sleep (Section 2.4). The alternate sleep phases, NREM sleep and shorter episodes of REM sleep, cycle in an ultradian manner. The metabolism and body temperature are variable during sleep. The lowering of brain temperature prior to sleep onset or when transitioning to NREM sleep is only 0.2–0.4 °C, with the temperature rising upon entering REM sleep or waking [200]. During REM sleep, thermoregulatory control is suspended. Hence, there are indications that redox–bioenergetics–temperature regulatory processes have a role not only in diurnal rhythms but also in ultradian ones. Although there is overall greater use of fatty-acid β-oxidation during the sleep phase, there is increased glycolysis and TCA cycling during REM sleep compared to NREM sleep (Section 2.4) [65]. On the basis that octanoic acid blocks glycolysis and is mitoprotective [201], the possibility of preferential mtFAS activity during an individual sleep phase or stage is worth considering (Section 3.4 and Section 6.4).

### 5.5. Torpor and Hibernation

Changes in redox–bioenergetics–temperature regulation during the sleep–wake cycle are an integral part of involuntary torpor and estivation, and voluntary hibernation. These hypometabolic/hypothermic conditions lasting short and long periods, respectively, allow small endotherms especially mammals to fast, conserve energy, reduce risk of predation, and generally survive winter months. The GSH:GSSG redox state in ground squirrel intestines was fivefold lower, and hence more oxidized, during hibernation season compared with summer active conditions [202]. In a similar but more exaggerated manner than experienced during sleep, UCP1/3 are severalfold more active in the mitochondria of hibernating, hypothermic Arctic ground squirrels than during euthermia [203]. Unlike UCP1 in brown adipose tissue, tellingly the UCP homologues, UCP2 and UCP3 (expressed in many tissue types and muscle, respectively) do not participate in non-shivering thermogenesis, with ROS mediation being a possible primary role.

The third component of redox–bioenergetics–temperature cycling, thermoregulation is intimately linked to redox and respiratory regulation during torpor and hibernation. Thirteen-lined ground squirrels hibernate in cycles of 1–3 weeks duration interspersed with short, ~24 h periods of arousal, euthermia, and sleep [204]. When the body temperature of this squirrel drops to 5 °C, its whole-body metabolic rate is only ~5% of the basal metabolic rate during euthermia at 37 °C [205]. Compared with euthermia, a reduced body temperature of 25 °C leads to promotion of ATP-maximizing fatty-acid oxidation, an inhibition of energy-expensive protein synthesis, reduction in mitochondria respiration, and enhanced proton leak production (Section 4.3) [206]. Reduction in the ambient temperature of isolated skeletal muscle mitochondria from thirteen-lined ground squirrels, with these mitochondria fueled by succinate (a CII substrate), increased the maximal ROS production [204]. Notwithstanding, mammals adopting low metabolic rates and mitoprotective adaptations during hibernation results in slowing their rates of senescence and increasing maximum life spans [207]. In summary, although sleep and hibernation have differing physiological processes, there are similarities in the differential redox, respiratory, and thermal regulatory response and the complementary lower metabolic rate and mitoprotective properties of both hibernation and sleep, which differ from the properties of wakefulness.

### 5.6. Hazards of Spaceflight and Space Radiation

Mitochondrial dysfunction, circadian rhythm disruption, and space radiation are hazards of spaceflight, but also have direct associations with our interactive redox–bioenergetics–temperature and differential mitochondrial–nuclear regulatory hypothesis. Some health issues of astronauts aboard the International Space Station are readily linked to microgravity, a space hazard linked to bone and muscle mass loss. However, space travel is also associated with adverse health issues of the central nervous system, immune system, cardiovascular tissues and particularly of the eye (e.g., cataracts and spaceflight-associated neuro-ocular syndrome), the increased risk of which are linked to the involvement of a variety of space hazards such as space radiation and circadian rhythm disturbances [142,208]. Ionizing radiation activates HSPs and adaptive responses such as nuclear DNA repair [144]. The radiolytic products of ionizing radiation generate calcium ions, Ca^2+^, in the endothelial reticulum, whose influx into mitochondria produces excessive ROS [209]. Perhaps relevant to space radiation health effects, there is experimental evidence using cell lines that the normal entry of Ca^2+^ into mitochondria is necessary to maintain the oscillatory nature of mitochondrial respiration [210].

Cell-free mtDNA, a biomarker of inflammation, oxidative stress and DNA damage, was measured in the plasma of 14 astronauts 10 days before the launch of brief flights, and measured again one or three days after returning from the International Space Station and found to be hugely elevated (~2- to 355-fold) [211]. Substantiation of spaceflight causing harmful effects to mitochondria was obtained from the analysis of multi-omics of 59 astronauts utilizing NASA’s GeneLab database [212]. The redoxomes of astronauts show disturbance. Astronauts’ GSH levels and GSH:GSSG ratio are both lowered in the liver [213], probably in part due to space radiation as radiotherapy X-rays elevates GSSG levels in blood [214]. Anion-mediators and cortisol are increased in astronauts [213] and melatonin decreased [208]. For unknown reasons, the thermoregulation of astronauts is disturbed, with astronauts developing over 2.5 months a persistently high body temperature of 1 °C above the normal value of 37 °C [215]. On the one hand, high doses of X-rays increase mitochondrial remodeling, fusion, and ETC supercomplexes, allowing moderate cellular adaptation and mitochondrial restoration [216]. On the other hand, restitution is compromised by spaceflight being associated with major metabolic and circadian disturbances [208]. For example, the perturbed circadian rhythms of astronauts prevent restoration of the immune system causing a decrease in nearly all their immune populations [213]. All-in-all there is much evidence that spaceflight disturbs all components of redox–bioenergetics–temperature cycling in astronauts with significant health consequences for long-duration missions.

## 6. Sleep Theories

### 6.1. Introduction

**Figure 3:**
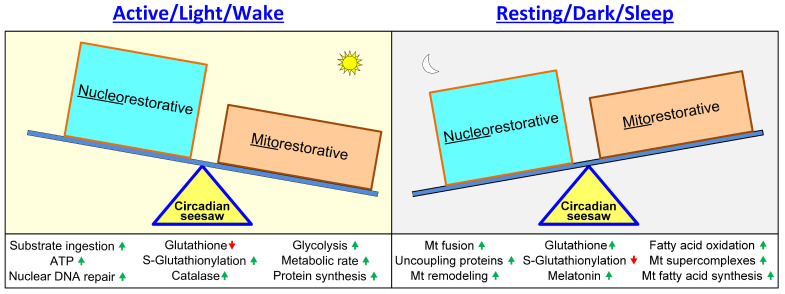
A circadian seesaw model illustrating how various cellular and mitochondrial (Mt) processes affect nucleorestorative and mitorestorative phases of the sleep–wake cycle. Process activity is shown raised 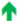 or lowered 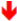.

Sleep, omnipresent among endothermic mammals and birds, ectothermic reptiles and amphibians, and even simpler animals, is a multifactorial physiological adaptation. There are many substantial theories of sleep, but also many unresolved issues [1]. Two sleep theories mentioned here, the energy conservation and free radical flux theories, are among those current theories for which our redox–bioenergetics–temperature and differential mitochondrial–nuclear regulatory hypothesis provides supporting detail. Nevertheless, it is unlikely that systemic energy conservation, or indeed cerebral free radical flux control, are the primary purposes of sleep [200]. This section makes the case for interactive redox–bioenergetics–temperature regulation being an important facet controlling the differential activities of sleep–wake cycling that especially benefits the brain at the tissue/organ level and mitochondria at the cellular level (Figure 3).

### 6.2. Energy Conservation and Free Radical Flux Theories

Energy savings as high as 30% during the resting/sleep phase is estimated, factoring in reductions in vigilance, motor activity, oxygen consumption rate, blood/tissue oxygenation, core temperature, and protein synthesis [8,9,62]. Three components that contribute most to ATP-consuming reactions and the resting metabolic rate are the proton leak in mitochondria producing heat (Section 3.2), maintaining sodium–potassium pumping, and protein synthesis (or mRNA translation) [217]. Protein synthesis and RNA/DNA synthesis are the greatest ATP-consumers and as such are the most sensitive to energy supply and are maximized after feeding during the wake phase [57,58,218]. Sodium–potassium pumping enzymes and some protein synthesis pathways are regulated by protein posttranslational modifiers including being inhibited by redox sensitive S-glutathionylation [50,219]. Thermal and oxidative distress in mitochondria can cause their dysfunction invoking a cellular integrated stress response, modifying the nuclear transcriptome and slowing down protein synthesis in the cytoplasm [220]. The depletion of GSH or an increase in GSSG may also lead to some protein S-glutathionylation inhibition of protein synthesis [221]. The synthesis of one molecule of GSH requires 2 ATP molecules. Mitochondrial dynamics regulate cellular GSH levels and its redox state [222]. During sleep, mitochondrial fusion and fatty-acid β-oxidation provide the conditions for mitochondria maintenance and efficient ATP production. However, energy conservation during sleep may prioritize GSH synthesis and limit protein synthesis.

Especially pertinent to the redox–bioenergetics–temperature and differential mitochondrial–nuclear regulatory hypothesis is the free radical flux theory of sleep by Reimund [223], which states that cerebral free radicals accumulated during wakefulness are neutralized during sleep by generated antioxidants. The high metabolic rate of the wake phase means that both mitochondrial ETC and non-ETC sources of ROS are at their maximum activity while awake (Section 2.4). There is evidence that, to counteract the ROS, enzymatic antioxidants usually peak in humans during the light phase, whereas most nonenzymatic antioxidants peak during the dark phase (Section 1 and Figure 2). Free radicals have short lifetimes, usually ≤ 1 μs [209], yet the detrimental effects of oxidative/nitrosative distress, and especially redox modifications, accumulate diurnally during the high-metabolic-rate wake phase. Going back to the example at the beginning of this article, mice sleep about four times longer than elephants. Based on the mass of mice (~20 g) and elephants (~2 tonne) the ratio of their higher mass-specific metabolic rate (which is proportional to mass^−1/4^) is about 18:1. One could speculate that this relatively higher rate puts more oxidative stress on the mitochondria of mice, and hence they have a greater need for more mitorestorative time asleep.

### 6.3. Protective and Restorative Aspects of the Sleep–Wake Cycle

#### 6.3.1. Neuroprotective Aspects of Sleep

Sleep is recognized as being neuroprotective and neurorestorative [224]. A well-known example is the observation that misfolded proteins such as amyloid-β (Aβ), present in the interstitial space surrounding cells in the brain, are mostly removed during sleep from the central nervous system by the glymphatic system, a perivascular network with astroglia end-feet [225]. Additional benefits that sleep bring to the brain are derived indirectly from observations on sleep deprivation. A remarkable study by Piéron [226] in the early 20th century of cerebrospinal fluid taken from sleep-deprived dogs and injected into nondeprived dogs found that it caused these recipient dogs to display behavioral signs of sleep deprivation [2]. GSSG accumulated in the waking state was later identified in the brain stem of sleep-deprived rats as an “endogenous sleep substance” [227]. Similarly, short-term sleep-loss experiments in animal models temporarily decrease the GSH:GSSG ratio in brain regions, indicating some oxidative stress, whereas long-term sleep loss causes greater oxidative changes, and probably permanent changes in this redox couple ratio [2]. Section 2.5 describes the restoration of redox couples GSH:GSSG and NADPH:NADP^+^ to higher ratio values, representative of more reduced conditions, as occur during adequate sleep. These examples demonstrate that sleep reduces biomarkers of excessive oxidative stress and restores normal redox states, especially in the brain.

#### 6.3.2. Nuclear DNA Repair during Wakefulness

Repair systems of oxidative damage to DNA are generally ATP-dependent [228]. High levels of ATP production, glycolytic metabolic products, protein synthesis, protein repair, and protein re-synthesis occur during the wake phase, differentially benefitting cellular and nuclear restitution (Section 2.1). Under normal diurnal conditions, the heat shock response appears to enable the repair of damage to proteins and nuclear DNA during the wake phase (Section 4.2). This circadian zenith of the activity of DNA response proteins may vary between the brain and nonbrain organs. Poly(ADP-ribose) polymerase 1 (PARP1) has an important role in many nuclear DNA repair processes, including oxidative DNA damage response initiator and participation in the heat shock response. Cell-cycle-regulated PARP1 dependent repair of DNA damage is tightly coupled to feeding and NAD^+^ enzymic activity [191,229,230]. Energy-intensive (especially by aerobic glycolysis) and biomass-intensive cell division is required for growth and replenishing dead cells but also is an opportunity for DNA repair. Experiments using the mitotic liver and other cells of mice demonstrate the coupling of organ cells circadian-regulated by peripheral clocks and cell-cycle dependent DNA repair [229,231].

Of the various nuclear DNA processes, nucleotide excision repair constitutes 40% or more of total DNA lesions and removes bulky DNA lesions, the sole system neutralizing major ultraviolet photolesions [232]. Nucleotide excision repair exhibits robust circadian oscillation in mouse liver, brain and skin with elevated levels of excision activity during the light phase, the time of maximal exposure to sunlight [233,234]. Base excision repair also is a major process maintaining nuclear (and mitochondrial) genomic integrity of ~40% of total endogenous DNA adducts [232]. Base excision repair recognizes and eliminates damaged bases of single strand breaks in DNA or the less common, oxidized base lesion, 8-oxo-7,8-dihydroguanosine (8-oxoG). Base excision repair, as shown for 8-oxoG repair in human lymphocytes, also follows a circadian rhythm and is at a maximum during morning hours [235]. Relatively rare but very cytotoxic, double-strand breaks contribute < 0.1% of endogenous DNA adducts in general but increase with genotoxic stress such as caused by ionizing radiation [232,236]. Double-strand breaks accumulate during the wake phase in glycolysis-dependent, nonmitotic, neurons of the zebrafish dorsal pallium; however, these nuclear DNA lesions are repaired during sleep [237]. Hence, with the less frequent double-strand break repair being an exception, the energy-intensive activity of nuclear DNA repair mainly occurs during the wake phase.

#### 6.3.3. Mitochondrial Fusion and Restoration during Sleep

Consider the timeline of repair to mtDNA damage, which if not addressed can impair bioenergetics, raise ROS production and accelerate aging [238]. Mitochondrial fission and fusion have wake and sleep phase preferences, respectively (Section 3.3). Mitochondrial fission can separate damaged mtDNA from the normal mtDNA copies within a given mitochondrion by creating a newly formed pair of mitochondria with one of them having the damaged DNA alone. Mitophagy can then eliminate these defective mitochondria. S-glutathionylation has a role in regulating mitophagy, autophagy and apoptosis, by desensitizing-ROS-signaling and by inhibiting p53 [45,48]. Although the experimental evidence is from a limited number of organs derived from nocturnal animals, it appears that for diurnal creatures such as humans, cell death peaks in the early-wake phase, whereas autophagy (and perhaps mitophagy) peaks during sleep [239]. S-glutathionylation also promotes the formation of the mitochondrial hyperfused state when GSH pools are oxidized [240]. The sleep-hormone melatonin, besides being an anion carrier mediator, also preferentially promotes fusion during sleep, a mitochondrial dynamic state enabling efficient mitochondrial function, when rapid cristae membrane remodeling occurs (Section 3.3), [241]. Consequently, it is during sleep that the replenishment of damaged mitochondrial contents principally occurs.

Our analysis of redox–bioenergetics–temperature cycling indicates that wakefulness is more protective and restorative to the nucleus, whereas sleep is more protective and restorative to mitochondria (Figure 3). There is experimental evidence to support the differential sleep–wake separation of nuclear and mitochondrial activities and that nuclear DNA repair, protein synthesis, protein repair, protein resynthesis, apoptosis, and transcriptional reprogramming primarily occur in primates during the daytime [17,242]. However, sleep deprivation and sleep apnea experiments provide insights that an important benefit of sleep is to minimize mitochondrial dysfunction and mtDNA damage [180]. Sleep deficit imposed on *Drosophila* elevates mitochondrial ROS and oxidative distress in sleep-inducing neurons [243]. Similarly, sleep deprivation in rodents increases oxidative stress and nuclear DNA damage, as well as impairing the mitochondrial function of CI–III, demonstrating that the prime benefits of sleep are in mitorestoration [244].

#### 6.3.4. Mitochondrial Respiratory Protection Provided by UCPs

The cellular role of a complex entity such as UCP may be too constrained by being solely based on two of its many key characteristics, namely thermogenesis and ROS-mediation, as described by three hypotheses (Section 5.2) that detail the advantages of inhibiting the coupling between electron transport and ATP synthesis in terms of providing respiratory protection, decelerating aging, and optimizing maximum-respiratory-rate conditions. Other key UCP characteristics are promoting fatty-acid β-oxidation, preventing hypoglycemia (Section 3.2), and UCPs regulating and being regulated by redox and anion-carrier mediator signaling (Section 3.3).

Proton leaks via UCPs may have a circadian-related role in the ~30% energy conservation during sleep (e.g., lower heart rate and cardiac heat production). The ~1 °C human sleep–wake temperature change accounts for only ~13% less heat energy being needed during sleep [13]. Both the raising and lowering of core temperature during sleep can be expected to increase ROS production based on experiments with isolated mitochondria (Section 4.3), however there are additional factors to consider in vivo, including external triggers such as anion carrier mediators. Anion carriers such as UCPs in mammals are also regulated by mediators such as fatty acids, ROS, S-glutathionylation, thyroid hormones, melatonin, and cortisol (Section 3.3). Although it cannot be stated definitively, the temperature-dependent generation of ROS during sleep is dampened by UCPs, especially in organs of high basal metabolic rate, and as during sleep, there are likely fatty-acid-mediated enhancements of the UCP proton leaks [115,116], which lower both mitochondrial ROS production and oxidative phosphorylation efficiency. Perturbed circadian rhythms and greater oxidative/metabolic stress caused by sleep deprivation in animal models elevate the expression of UCPs in the liver and muscle [245]; this has the benefit of lower ROS production and maintained redox homeostasis but is costly in terms of less efficient ATP synthesis. We contend that a more comprehensive description of UCP mitochondrial transmembrane transporters, embracing some of the key attributes of previous hypotheses, is that UCPs have major reciprocal interactions with the redoxome, bioenergetics and temperature, which enable important mitochondrial functions of sleep–wake cycling.

### 6.4. Control of Protein Activity and Metabolic Futile Cycling Avoidance by Sleep–Wake Phases

A mitochondrial liver protein is nearly 5-fold (38÷8%) more likely to display circadian oscillations than a protein in the whole proteome, comparing the data from two independent studies (Section 2.1), [31,32]. Earlier it was also noted that only ~6% of the overall human proteome is located in mitochondria, yet this organelle hosts almost 30% of the altered cysteine sites in murine skeletal muscle (Section 2.4) [52]. Therefore, although these comparisons are based on independent studies of differing tissues and methods, it can be tentatively inferred that a mitochondrial protein is several-fold more likely to undergo circadian cysteine-mediated posttranslational changes than a protein in the non-mitochondrial cellular volume. Hence, mitochondria are over-represented cellularly in sleep–wake cycling especially involving reversible cysteine modifications, which are mainly inhibitive of protein activity with PSSG-activated mitoprotective exceptions such as ANT, HIF-1α, and CII (Section 2.4).

Sleep–wake cycling appears to provide the opportunity of temporal separation to prevent metabolic futile cycling. The greater provision of fatty-acid oxidation and ATP-maximizing oxidative phosphorylation during sleep is a special requirement of the immune system (Section 3.4). Futile cycles in various organisms have been observed to lower growth rate, elevate oxygen consumption, and raise H_2_O_2_ production [228]. Consequently, metabolic futile cycles can be prevented by transcriptional and allosteric regulation. We propose that an additional benefit of the sleep–wake cycles is the partial avoidance of futile cycling by temporal separation, which applies to the Randle cycle and possibly the cycling of mtFAS and fatty-acid β-oxidation (Section 3.4). In addition, it is proposed that the attributes, such as ROS, heat and ATP production, of mitochondrial futile proton cycling are temporally controlled in a circadian/ultradian manner. In summary, many reasons support redox–bioenergetics–temperature regulatory control being an essential element of the sleep–wake cycle allowing differential mitochondrial/nuclear activities with sleep being a restorative phase for mitochondria.

## 7. Testing of the Hypothesis, and Conclusions

There are important implications of mitochondrial–nuclear coevolution and coregulation in eukaryote cells [246]. Sustaining the strong rhythmic interactions between cellular and especially mitochondrial redox-controlled posttranslational modifications, redox couples, respiratory uncoupling, macrometabolics, and thermoregulation are necessary to preserve the differential properties of the sleep–wake cycles and maintain good health (Figure 1). Reported experimental evidence provided in support of the redox–bioenergetics–temperature and differential mitochondrial–nuclear regulatory hypothesis has been collected from published human and animal studies. We acknowledge that our hypothesis is not a substitute for data derived from carefully constructed and well-controlled experimental studies, and future studies may not confirm some of the data described and conclusions reached. We also acknowledge that no single hypothesis or theory, including our differential mitochondrial–nuclear hypothesis, can comprehensively describe the complexities of sleep–wake cycling, let alone sleep phases and stages.

There are limitations when elaborating upon these reported findings on the sleep–wake cycle due to the vagaries of experimental reproducibility and variations related to species, organ or cell type. Notwithstanding, most reliance in developing the hypothesis has been placed on human, whole body-, organ-, tissue-, or mitochondria-specific representative measurements such as plasma constituents, core temperature, RQ and redox parameters. Therefore, confirmation of the hypothesis and the role of tripartite-interactome signaling (Figure 1) and differential mitochondrial–nuclear activities (Figure 2 and Figure 3) would benefit from consecutive measurements in humans where possible, including the longitudinal effects of aging and the diurnal rhythmic interaction of S-glutathionylation and S-nitrosylation of proteins, UCPs, mitochondrial dynamics, and thermal/oxidative stress parameters. In addition, confirmation is needed of a proposed over-representation of S-glutathionylation and S-nitrosylation of mitochondria proteins compared to the whole-cell proteome (Section 2.2 and Section 2.4). The examining of sleep phase or stage variations in mitochondrial biomolecules, such as UCPs, CII, TCA intermediates, GSH, and ^•^NO, is recommended and their potential effect on the temporal separation of mtFAS and fatty-acid β-oxidation. Better knowledge of these circadian and ultradian sleep–wake cycles, besides providing evidence on the validity of the proposed interactions, would further enlighten how aiding the maintenance of these interactive processes could mitigate related issues of aging and certain diseases.

An example where a better understanding of the differential mitochondrial–nuclear activities of sleep–wake cycling could improve health outcomes is in SIDS. It would appear from our analysis that an inadequate perinatal maturation of the circadian rhythm during this critical development period is an important facet of the syndrome. Therefore, further measures to better entrain the sleep–wake rhythm beyond the successful adoption of the supine, face-up position, such as light therapy, thermoregulation, feeding and other behavior adjustments to enhance the newborn’s diurnal pattern [163], are strongly supported by our circadian-related hypothesis.

In conclusion, considerable cellular resources and many redox–bioenergetics–temperature regulatory processes are involved in controlling spatially and temporally the physiological, metabolic, protective and restorative mechanisms of sleep–wake cycling, which is beneficial to humans’ health and longevity. The redoxome plays an integral role in regulating the circadian clock through spatiotemporal fluctuations in the mitochondrial interactome, including quantitative variation of ROS, GSH, GSSG, UCPs, CII and the redox-reversible posttranslational modifications of metabolic proteins. The redox–bioenergetics–temperature and differential mitochondrial–nuclear regulatory hypothesis places reversible cysteine-mediated redox signaling, UCPs and substrate cycles at the center of providing strong physiological and metabolic differentiation during sleep–wake cycling, including the temporal separation of potential futile cycling of metabolic pathways. We also contend that maintenance of the spatiotemporal fluctuations of the redoxome, bioenergetics, and thermoregulation promotes nucleorestorative wakefulness and mitorestorative sleep.

## Figures and Tables

**Table 1:** Mitochondrial redox proteins, namely complexes I–V (CI–V) of the electron transport chain (ETC, in blue text) and selected non-ETC enzymes described in terms of reactive oxygen species (ROS) production, protein S-glutathionylation (PSSG), S-nitrosylation (SNO), modifications and circadian oscillations. The reports are for various mammalian tissues/organs and species.

Mitochondrial Proteins	Protein Function	Abbreviation [ROS Emitting]	^a^ ROS Inhibiting (Anti-ROS) or Redox Sensor	^b^ Redox Inhibited or [Activated]	^c^ Circadian Regulated	References
Complex I (Ubiquinone oxidoreductase)	ETC enzyme, oxidizes NADH from TCA cycle	CI [most ETC ROS]	✓ redox sensor	✓ PSSG, ✓ SNO	✓	^a^ [22], ^b^ [76], ^b^ [77], ^b^ [78], ^c^ [32]
Complex II (Succinate dehydrogenase)	ETC enzyme, oxidizes succinate in TCA cycle	CII [little ETC ROS]		✓ PSSG [activated], ✓ SNO	✓	^b^ [79], ^c^ [80]
Complex III (Coenzyme Q)	ETC enzyme	CIII [some ETC ROS]		✓ PSSG (via MIA40), ✓ SNO	✓ (& via MIA40)	^b^ [81], ^c^ [32]
Complex IV (Cytochrome C oxidase)	ETC enzyme	CIV		✓ PSSG (& via MIA40), ✓ SNO	✓ (& via MIA40)	^b^ [47], ^b^ [81], ^c^ [32]
Complex V (ATP synthase)	ETC enzyme	CV		✓ PSSG, ✓ SNO	✓	^b^ [72], ^c^ [82], ^c^ [32]
Adenine nucleotide translocase	ADP/ATP exchange	ANT	✓ Reverses apoptosis process	✓ PSSG [activated], ✓ SNO	✓ based on ADP/ATP ratio	^a,b^ [60], ^c^ [83]
α-ketoglutarate dehydrogenase	Lipoate TCA cycle catalysis of substrates	^d, f^ α-KGDH or OGDH [ROS 2 x CI]	✓ redox sensor	✓ PSSG (10 sites), ALA, ✓ SNO	✓	^b^ [84], ^c^ [32]
Branched-chain keto acid dehydrogenase	Rate-limiting enzyme catabolizing α-ketoacids	^d, f^ BCKDH [ROS 8 x CI]		✗ non-PSSG, ALA	✓	^b^ [55], ^c^ [56]
Dynamin-related protein-1	Pro-fission protein	DRP1		✗ non-PSSG, ✓ SNO	✓	^b^ [85], ^c^ [86], ^c^ [87]
Glutaredoxin-2	GSSG reducing enzyme using GSH as cofactor	GRX2	✓ anti-ROS, Fe-S cluster redox sensor	✗ non-PSSG, reverses PSSG reactions	✓ based on GSH:GSSG	^a^ [88], ^a^ [48], ^c^ [24]
Peroxiredoxin III	Hydrogen peroxide-scavenging enzyme	^e^ PRXIII	✓ anti-ROS, redox sensor	✓ S-sulfinylation	✓	^b^ [89], ^c^ [16], ^c^ [90]
Pyruvate dehydrogenase	Lipoate enzyme in glycolysis, pyruvate to acetyl-CoA pathway	^d, f^ PDH [ROS 4 x CI]	✓ redox sensor	✓ PSSG, ALA, ✓ SNO	✓	^b^ [84], ^c^ [32], ^c^ [91]
Uncoupling proteins	Transports protons across the mitochondrial inner membrane	UCP2/3	✓ anti-ROS, redox sensor	✓ PSSG [activated]	✓	^a^ [92], ^b^ [93], ^b^ [94], ^c^ [95], ^c^ [96]

^a,b,c^ Refers to the references given in the last column of the table. ^d^ Dehydrogenases regulated by ROS, mediated by disulfide bond of the lipoyl moiety. ^e^ Hyperoxidized to PRXIII-SO_2_. ^f^ See reference [54]. Abbreviations: ALA, α-lipoic acid; ETC, electron transport chain; Fe-S cluster, iron–sulfur cluster; GSH, glutathione; GSSG, glutathione disulfide; MIA40, mitochondrial intermembrane space assembly 40; TCA cycle, tricarboxylic acid cycle.

## Abbreviations

α-KGDH	α-ketoglutarate dehydrogenase
acetyl-CoA	acetyl coenzyme A
ADP	adenosine diphosphate
ANT	adenine nucleotide translocase
ATP	adenosine triphosphate
BCKDH	branched-chain keto acid dehydrogenase
CI–V	mitochondrial complexes I–V
DRP1	dynamin-related protein 1
ETC	electron transport chain
FAS	fatty-acid synthesis
GPX	glutathione peroxidase
GRX	glutaredoxin
GSH	glutathione
GSSG	glutathione disulfide
H_2_O_2_	hydrogen peroxide
HIF-1	hypoxia-inducible factor 1
HSF1	heat shock factor 1
HSP	heat stress protein
mtDNA	mitochondrial DNA
mtFAS	mitochondrial fatty-acid synthesis
mTOR	mammalian target of rapamycin
mTORC1	mTOR complex 1
NAD^+^	nicotinamide adenine dinucleotide
NADH	reduced NAD^+^
NADP^+^	nicotinamide adenine dinucleotide phosphate
NADPH	reduced NADP^+^
^•^NO	nitric oxide
NOS	nitric oxide synthase
NREM	non-rapid eye movement
O_2_	molecular oxygen
O_2_^•−^	superoxide radical
^•^OH	hydroxyl radical
ONOO^−^	peroxynitrite
PDH	pyruvate dehydrogenase
PRX	peroxiredoxin
PSSG	protein S-glutathionylation
REM	rapid eye movement
ROOR	peroxide functional group
ROS/RNS/RSS	reactive oxygen/nitrogen/sulfur species
RQ	respiratory quotient
SCN	suprachiasmatic nucleus
SIDS	sudden infant death syndrome
SIRT3	sirtuin 3
SOD	superoxide dismutase
SRX	sulfiredoxin
T_3_	triiodothyronine
T_4_	thyroxine
TCA cycle	tricarboxylic acid cycle
TRX	thioredoxin
TSH	thyroid-stimulating hormone
UCP	uncoupling protein

## References

[1] Rechtschaffen A. (1998). Current perspectives on the function of sleep. Perspect. Biol. Med..

[2] Villafuerte G., Miguel-Puga A., Rodriguez E.M., Machado S., Manjarrez E., Arias-Carrion O. (2015). Sleep deprivation and oxidative stress in animal models: A systematic review. Oxid. Med. Cell. Longev..

[3] Harman D. (2003). The free radical theory of aging. Antioxid. Redox Signal..

[4] Finkel T., Holbrook N.J. (2000). Oxidants, oxidative stress and the biology of ageing. Nature.

[5] Rubner M. (1908). Das Problem der Lebensdaur und seine Beziehungen zu Wachstum und Ernärhung.

[6] Pearl R. (1928). The Rate of Living, Being an Account of Some Experimental Studies on the Biology of Life Duration.

[7] Savage V.M., West G.B. (2007). A quantitative, theoretical framework for understanding mammalian sleep. Proc. Natl. Acad. Sci. USA.

[8] Schmidt M.H., Swang T.W., Hamilton I.M., Best J.A. (2017). State-dependent metabolic partitioning and energy conservation: A theoretical framework for understanding the function of sleep. PLoS ONE.

[9] Zitting K.M., Vujovic N., Yuan R.K., Isherwood C.M., Medina J.E., Wang W., Buxton O.M., Williams J.S., Czeisler C.A., Duffy J.F. (2018). Human resting energy expenditure varies with circadian phase. Curr. Biol..

[10] Stritesky Larssen K., Lyberg T. (2006). Oxidative status—Age- and circadian variations?—A study in leukocytes/plasma. Neuro Endocrinol. Lett..

[11] Landsberg L., Young J.B., Leonard W.R., Linsenmeier R.A., Turek F.W. (2009). Do the obese have lower body temperatures? A new look at a forgotten variable in energy balance. Trans. Am. Clin. Climatol. Assoc..

[12] Tan C.L., Knight Z.A. (2018). Regulation of body temperature by the nervous system. Neuron.

[13] Du Bois E.F. (1921). The basal metabolism in fever. J. Am. Med. Assoc..

[14] Refinetti R., Menaker M. (1992). The circadian rhythm of body temperature. Physiol. Behav..

[15] Ali S.S., Marcondes M.C., Bajova H., Dugan L.L., Conti B. (2010). Metabolic depression and increased reactive oxygen species production by isolated mitochondria at moderately lower temperatures. J. Biol. Chem..

[16] Milev N.B., Rhee S.G., Reddy A.B. (2018). Cellular timekeeping: It’s redox o’clock. Cold Spring Harb. Perspect. Biol..

[17] Mure L.S., Le H.D., Benegiamo G., Chang M.W., Rios L., Jillani N., Ngotho M., Kariuki T., Dkhissi-Benyahya O., Cooper H.M. (2018). Diurnal transcriptome atlas of a primate across major neural and peripheral tissues. Science.

[18] Torrence M.E., MacArthur M.R., Hosios A.M., Valvezan A.J., Asara J.M., Mitchell J.R., Manning B.D. (2021). The mTORC1-mediated activation of ATF4 promotes protein and glutathione synthesis downstream of growth signals. elife.

[19] Plano S.A., Baidanoff F.M., Trebucq L.L., Suarez S.A., Doctorovich F., Golombek D.A., Chiesa J.J. (2021). Redox and antioxidant modulation of circadian rhythms: Effects of nitroxyl, N-acetylcysteine and glutathione. Molecules.

[20] Rolfe D.F., Hulbert A.J., Brand M.D. (1994). Characteristics of mitochondrial proton leak and control of oxidative phosphorylation in the major oxygen-consuming tissues of the rat. Biochim. Biophys. Acta.

[21] Sies H. (2017). Hydrogen peroxide as a central redox signaling molecule in physiological oxidative stress: Oxidative eustress. Redox Biol..

[22] Murphy M.P. (2009). How mitochondria produce reactive oxygen species. Biochem. J..

[23] Singh R.B., Niaz M.A., Cornelissen G., Otsuka K., Siegelová J., Fišer B., Halberg F. (2001). Circadian rhythmicity of circulating vitamin concentrations. Scripta Med..

[24] Blanco R.A., Ziegler T.R., Carlson B.A., Cheng P.Y., Park Y., Cotsonis G.A., Accardi C.J., Jones D.P. (2007). Diurnal variation in glutathione and cysteine redox states in human plasma. Am. J. Clin. Nutr..

[25] Wilking M., Ndiaye M., Mukhtar H., Ahmad N. (2013). Circadian rhythm connections to oxidative stress: Implications for human health. Antioxid. Redox Signal..

[26] Gordon C.J. (2017). The mouse thermoregulatory system: Its impact on translating biomedical data to humans. Physiol. Behav..

[27] Jezek P., Holendova B., Garlid K.D., Jaburek M. (2018). Mitochondrial uncoupling proteins: Subtle regulators of cellular redox signaling. Antioxid. Redox Signal..

[28] Jastroch M., Divakaruni A.S., Mookerjee S., Treberg J.R., Brand M.D. (2010). Mitochondrial proton and electron leaks. Essays Biochem..

[29] Chance B., Sies H., Boveris A. (1979). Hydroperoxide metabolism in mammalian organs. Physiol. Rev..

[30] Balaban R.S., Nemoto S., Finkel T. (2005). Mitochondria, oxidants, and aging. Cell.

[31] Wang Y., Song L., Liu M., Ge R., Zhou Q., Liu W., Li R., Qie J., Zhen B., Wang Y. (2018). A proteomics landscape of circadian clock in mouse liver. Nat. Commun..

[32] Neufeld-Cohen A., Robles M.S., Aviram R., Manella G., Adamovich Y., Ladeuix B., Nir D., Rousso-Noori L., Kuperman Y., Golik M. (2016). Circadian control of oscillations in mitochondrial rate-limiting enzymes and nutrient utilization by PERIOD proteins. Proc. Natl. Acad. Sci. USA.

[33] Robles M.S., Humphrey S.J., Mann M. (2017). Phosphorylation is a central mechanism for circadian control of metabolism and physiology. Cell Metab..

[34] Hebert A.S., Dittenhafer-Reed K.E., Yu W., Bailey D.J., Selen E.S., Boersma M.D., Carson J.J., Tonelli M., Balloon A.J., Higbee A.J. (2013). Calorie restriction and SIRT3 trigger global reprogramming of the mitochondrial protein acetylome. Mol. Cell.

[35] Townsend D.M., Lushchak V.I., Cooper A.J. (2014). A comparison of reversible versus irreversible protein glutathionylation. Adv. Cancer Res..

[36] Putker M., O’Neill J.S. (2016). Reciprocal control of the circadian clock and cellular redox state—A critical appraisal. Mol. Cells.

[37] Grek C.L., Zhang J., Manevich Y., Townsend D.M., Tew K.D. (2013). Causes and consequences of cysteine S-glutathionylation. J. Biol. Chem..

[38] Canals S., Casarejos M.J., de Bernardo S., Rodriguez-Martin E., Mena M.A. (2003). Nitric oxide triggers the toxicity due to glutathione depletion in midbrain cultures through 12-lipoxygenase. J. Biol. Chem..

[39] Foster M.W., Hess D.T., Stamler J.S. (2009). Protein S-nitrosylation in health and disease: A current perspective. Trends Mol. Med..

[40] Piantadosi C.A. (2012). Regulation of mitochondrial processes by protein S-nitrosylation. Biochim. Biophys. Acta.

[41] Doulias P.T., Tenopoulou M., Greene J.L., Raju K., Ischiropoulos H. (2013). Nitric oxide regulates mitochondrial fatty acid metabolism through reversible protein S-nitrosylation. Sci. Signal..

[42] Cespuglio R., Amrouni D., Meiller A., Buguet A., Gautier-Sauvigne S. (2012). Nitric oxide in the regulation of the sleep-wake states. Sleep Med. Rev..

[43] Golombek D.A., Agostino P.V., Plano S.A., Ferreyra G.A. (2004). Signaling in the mammalian circadian clock: The NO/cGMP pathway. Neurochem. Int..

[44] Rodrigo G.C., Herbert K.E. (2018). Regulation of vascular function and blood pressure by circadian variation in redox signalling. Free Radic. Biol. Med..

[45] Sun R., Eriksson S., Wang L. (2012). Oxidative stress induced S-glutathionylation and proteolytic degradation of mitochondrial thymidine kinase. J. Biol. Chem..

[46] Townsend D.M., Manevich Y., He L., Hutchens S., Pazoles C.J., Tew K.D. (2009). Novel role for glutathione S-transferase π: Regulator of protein S-glutathionylation following oxidative and nitrosative stress. J. Biol. Chem..

[47] Fratelli M., Demol H., Puype M., Casagrande S., Eberini I., Salmona M., Bonetto V., Mengozzi M., Duffieux F., Miclet E. (2002). Identification by redox proteomics of glutathionylated proteins in oxidatively stressed human T lymphocytes. Proc. Natl. Acad. Sci. USA.

[48] Mailloux R.J., Gill R., Young A. (2020). Protein S-glutathionylation and the regulation of cellular functions. Oxidative Stress: Eustress and Distress.

[49] Townsend D.M. (2007). S-Glutathionylation: Indicator of cell stress and regulator of the unfolded protein response. Mol. Interv..

[50] Duan J., Kodali V.K., Gaffrey M.J., Guo J., Chu R.K., Camp D.G., Smith R.D., Thrall B.D., Qian W.J. (2016). Quantitative profiling of protein S-glutathionylation reveals redox-dependent regulation of macrophage function during nanoparticle-induced oxidative stress. ACS Nano.

[51] Lind C., Gerdes R., Hamnell Y., Schuppe-Koistinen I., von Lowenhielm H.B., Holmgren A., Cotgreave I.A. (2002). Identification of S-glutathionylated cellular proteins during oxidative stress and constitutive metabolism by affinity purification and proteomic analysis. Arch. Biochem. Biophys..

[52] Campbell M.D., Duan J., Samuelson A.T., Gaffrey M.J., Merrihew G.E., Egertson J.D., Wang L., Bammler T.K., Moore R.J., White C.C. (2019). Improving mitochondrial function with SS-31 reverses age-related redox stress and improves exercise tolerance in aged mice. Free Radic. Biol. Med..

[53] Mailloux R.J. (2020). An update on mitochondrial reactive oxygen species production. Antioxidants.

[54] Quinlan C.L., Goncalves R.L., Hey-Mogensen M., Yadava N., Bunik V.I., Brand M.D. (2014). The 2-oxoacid dehydrogenase complexes in mitochondria can produce superoxide/hydrogen peroxide at much higher rates than complex I. J. Biol. Chem..

[55] Hirschenson J., Mailloux R.J. (2021). The glutathionylation agent disulfiram augments superoxide/hydrogen peroxide production when liver mitochondria are oxidizing ubiquinone pool-linked and branched chain amino acid substrates. Free Radic. Biol. Med..

[56] Wohlhueter R.M., Harper A.E. (1970). Coinduction of rat liver branched chain α-keto acid dehydrogenase activities. J. Biol. Chem..

[57] Clugston G.A., Garlick P.J. (1982). The response of protein and energy metabolism to food intake in lean and obese man. Hum. Nutr. Clin. Nutr..

[58] Jouffe C., Cretenet G., Symul L., Martin E., Atger F., Naef F., Gachon F. (2013). The circadian clock coordinates ribosome biogenesis. PLoS Biol..

[59] Young A., Gill R., Mailloux R.J. (2019). Protein S-glutathionylation: The linchpin for the transmission of regulatory information on redox buffering capacity in mitochondria. Chem. Biol. Interact..

[60] Queiroga C.S., Almeida A.S., Martel C., Brenner C., Alves P.M., Vieira H.L. (2010). Glutathionylation of adenine nucleotide translocase induced by carbon monoxide prevents mitochondrial membrane permeabilization and apoptosis. J. Biol. Chem..

[61] Jeon D., Park H.J., Kim H.S. (2018). Protein S-glutathionylation induced by hypoxia increases hypoxia-inducible factor-1α in human colon cancer cells. Biochem. Biophys. Res. Commun..

[62] Adamovich Y., Ladeuix B., Sobel J., Manella G., Neufeld-Cohen A., Assadi M.H., Golik M., Kuperman Y., Tarasiuk A., Koeners M.P. (2019). Oxygen and carbon dioxide rhythms are circadian clock controlled and differentially directed by behavioral signals. Cell Metab..

[63] Pfleger J., He M., Abdellatif M. (2015). Mitochondrial complex II is a source of the reserve respiratory capacity that is regulated by metabolic sensors and promotes cell survival. Cell. Death Dis..

[64] Bose H.S., Marshall B., Debnath D.K., Perry E.W., Whittal R.M. (2020). Electron transport chain complex II regulates steroid metabolism. iScience.

[65] Nowak N., Gaisl T., Miladinovic D., Marcinkevics R., Osswald M., Bauer S., Buhmann J., Zenobi R., Sinues P., Brown S.A. (2021). Rapid and reversible control of human metabolism by individual sleep states. Cell Rep..

[66] Stepanova A., Shurubor Y., Valsecchi F., Manfredi G., Galkin A. (2016). Differential susceptibility of mitochondrial complex II to inhibition by oxaloacetate in brain and heart. Biochim. Biophys. Acta.

[67] Schmidt M.H. (2014). The energy allocation function of sleep: A unifying theory of sleep, torpor, and continuous wakefulness. Neurosci. Biobehav. Rev..

[68] Wang T.A., Yu Y.V., Govindaiah G., Ye X., Artinian L., Coleman T.P., Sweedler J.V., Cox C.L., Gillette M.U. (2012). Circadian rhythm of redox state regulates excitability in suprachiasmatic nucleus neurons. Science.

[69] Khazim K., Giustarini D., Rossi R., Verkaik D., Cornell J.E., Cunningham S.E., Mohammad M., Trochta K., Lorenzo C., Folli F. (2013). Glutathione redox potential is low and glutathionylated and cysteinylated hemoglobin levels are elevated in maintenance hemodialysis patients. Transl. Res..

[70] Chatgilialoglu C., Ferreri C. (2021). Reductive stress of sulfur-containing amino acids within proteins and implication of tandem protein-lipid damage. Int. J. Mol. Sci..

[71] Schafer F.Q., Buettner G.R. (2001). Redox environment of the cell as viewed through the redox state of the glutathione disulfide/glutathione couple. Free Radic. Biol. Med..

[72] Garcia J., Han D., Sancheti H., Yap L.P., Kaplowitz N., Cadenas E. (2010). Regulation of Mitochondrial Glutathione Redox Status and Protein Glutathionylation by Respiratory Substrates. J. Biol. Chem..

[73] Mullarky E., Cantley L.C., Nakao K., Minato N., Uemoto S. (2015). Diverting glycolysis to combat oxidative stress. Innovative Medicine: Basic Research and Development.

[74] O’Neill J.S., Reddy A.B. (2011). Circadian clocks in human red blood cells. Nature.

[75] Rey G., Valekunja U.K., Feeney K.A., Wulund L., Milev N.B., Stangherlin A., Ansel-Bollepalli L., Velagapudi V., O’Neill J.S., Reddy A.B. (2016). The pentose phosphate pathway regulates the circadian clock. Cell Metab..

[76] Taylor E.R., Hurrell F., Shannon R.J., Lin T.K., Hirst J., Murphy M.P. (2003). Reversible glutathionylation of complex I increases mitochondrial superoxide formation. J. Biol. Chem..

[77] Xiong Y., Uys J.D., Tew K.D., Townsend D.M. (2011). S-Glutathionylation: From molecular mechanisms to health outcomes. Antioxid. Redox Signal..

[78] Gill R.M., O’Brien M., Young A., Gardiner D., Mailloux R.J. (2018). Protein S-glutathionylation lowers superoxide/hydrogen peroxide release from skeletal muscle mitochondria through modification of complex I and inhibition of pyruvate uptake. PLoS ONE.

[79] Chen Y.R., Chen C.L., Pfeiffer D.R., Zweier J.L. (2007). Mitochondrial complex II in the post-ischemic heart: Oxidative injury and the role of protein S-glutathionylation. J. Biol. Chem..

[80] Wielgus-Serafinska E., Plewka A., Kaminski M. (1993). Circadian variation of mitochondrial succinic dehydrogenase and microsomal cytochrome P-450 dependent monooxygenase activity in the liver of sexually immature and mature rats. J. Physiol. Pharmacol..

[81] Thiriveedi V.R., Mattam U., Pattabhi P., Bisoyi V., Talari N.K., Krishnamoorthy T., Sepuri N.B.V. (2020). Glutathionylated and Fe-S cluster containing hMIA40 (CHCHD4) regulates ROS and mitochondrial complex III and IV activities of the electron transport chain. Redox Biol..

[82] McCarthy J.J., Andrews J.L., McDearmon E.L., Campbell K.S., Barber B.K., Miller B.H., Walker J.R., Hogenesch J.B., Takahashi J.S., Esser K.A. (2007). Identification of the circadian transcriptome in adult mouse skeletal muscle. Physiol. Genomics.

[83] Sandbichler A.M., Jansen B., Peer B.A., Paulitsch M., Pelster B., Egg M. (2018). Metabolic plasticity enables circadian adaptation to acute hypoxia in zebrafish cells. Cell. Physiol. Biochem..

[84] O’Brien M., Chalker J., Slade L., Gardiner D., Mailloux R.J. (2017). Protein S-glutathionylation alters superoxide/hydrogen peroxide emission from pyruvate dehydrogenase complex. Free Radic. Biol. Med..

[85] Cho D.H., Nakamura T., Fang J., Cieplak P., Godzik A., Gu Z., Lipton S.A. (2009). S-Nitrosylation of Drp1 mediates beta-amyloid-related mitochondrial fission and neuronal injury. Science.

[86] Schmitt K., Grimm A., Dallmann R., Oettinghaus B., Restelli L.M., Witzig M., Ishihara N., Mihara K., Ripperger J.A., Albrecht U. (2018). Circadian control of DRP1 activity regulates mitochondrial dynamics and bioenergetics. Cell Metab..

[87] Ding M., Feng N., Tang D., Feng J., Li Z., Jia M., Liu Z., Gu X., Wang Y., Fu F. (2018). Melatonin prevents Drp1-mediated mitochondrial fission in diabetic hearts through SIRT1-PGC1α pathway. J. Pineal Res..

[88] Lillig C.H., Berndt C., Vergnolle O., Lonn M.E., Hudemann C., Bill E., Holmgren A. (2005). Characterization of human glutaredoxin 2 as iron-sulfur protein: A possible role as redox sensor. Proc. Natl. Acad. Sci. USA.

[89] Mailloux R.J., Jin X., Willmore W.G. (2014). Redox regulation of mitochondrial function with emphasis on cysteine oxidation reactions. Redox Biol..

[90] Kil I.S., Ryu K.W., Lee S.K., Kim J.Y., Chu S.Y., Kim J.H., Park S., Rhee S.G. (2015). Circadian oscillation of sulfiredoxin in the mitochondria. Mol. Cell.

[91] Reinke H., Asher G. (2019). Crosstalk between metabolism and circadian clocks. Nat. Rev. Mol. Cell Biol..

[92] Mailloux R.J., Harper M.E. (2011). Uncoupling proteins and the control of mitochondrial reactive oxygen species production. Free Radic. Biol. Med..

[93] Mailloux R.J., Seifert E.L., Bouillaud F., Aguer C., Collins S., Harper M.E. (2011). Glutathionylation acts as a control switch for uncoupling proteins UCP2 and UCP3. Biol. Chem..

[94] Mailloux R.J., Fu A., Robson-Doucette C., Allister E.M., Wheeler M.B., Screaton R., Harper M.E. (2012). Glutathionylation state of uncoupling protein-2 and the control of glucose-stimulated insulin secretion. J. Biol. Chem..

[95] Seshadri N., Jonasson M.E., Hunt K.L., Xiang B., Cooper S., Wheeler M.B., Dolinsky V.W., Doucette C.A. (2017). Uncoupling protein 2 regulates daily rhythms of insulin secretion capacity in MIN6 cells and isolated islets from male mice. Mol. Metab..

[96] Yasumoto Y., Stoiljkovic M., Kim J.D., Sestan-Pesa M., Gao X.B., Diano S., Horvath T.L. (2021). Ucp2-dependent microglia-neuronal coupling controls ventral hippocampal circuit function and anxiety-like behavior. Mol. Psychiatry.

[97] Skulachev V.P. (1996). Role of uncoupled and non-coupled oxidations in maintenance of safely low levels of oxygen and its one-electron reductants. Q. Rev. Biophys..

[98] Korshunov S.S., Skulachev V.P., Starkov A.A. (1997). High protonic potential actuates a mechanism of production of reactive oxygen species in mitochondria. FEBS Lett..

[99] Gerencser A.A., Chinopoulos C., Birket M.J., Jastroch M., Vitelli C., Nicholls D.G., Brand M.D. (2012). Quantitative measurement of mitochondrial membrane potential in cultured cells: Calcium-induced de- and hyperpolarization of neuronal mitochondria. J. Physiol..

[100] de Goede P., Wefers J., Brombacher E.C., Schrauwen P., Kalsbeek A. (2018). Circadian rhythms in mitochondrial respiration. J. Mol. Endocrinol..

[101] Porter R.K., Brand M.D. (1993). Body mass dependence of H^+^ leak in mitochondria and its relevance to metabolic rate. Nature.

[102] Brand M.D., Pakay J.L., Ocloo A., Kokoszka J., Wallace D.C., Brookes P.S., Cornwall E.J. (2005). The basal proton conductance of mitochondria depends on adenine nucleotide translocase content. Biochem. J..

[103] Dummler K., Muller S., Seitz H.J. (1996). Regulation of adenine nucleotide translocase and glycerol 3-phosphate dehydrogenase expression by thyroid hormones in different rat tissues. Biochem. J..

[104] Schönfeld P., Wojtczak L. (2007). Fatty acids decrease mitochondrial generation of reactive oxygen species at the reverse electron transport but increase it at the forward transport. Biochim. Biophys. Acta.

[105] Solmonson A., Mills E.M. (2016). Uncoupling proteins and the molecular mechanisms of thyroid thermogenesis. Endocrinology.

[106] Dulloo A.G., Samec S. (2001). Uncoupling proteins: Their roles in adaptive thermogenesis and substrate metabolism reconsidered. Br. J. Nutr..

[107] Ricquier D. (1999). Uncoupling protein-2 (UCP2): Molecular and genetic studies. Int. J. Obes. Relat. Metab. Disord..

[108] He M., Ma Y., Wang R., Zhang J., Jing L., Li P.A. (2020). Deletion of mitochondrial uncoupling protein 2 exacerbates mitochondrial damage in mice subjected to cerebral ischemia and reperfusion injury under both normo- and hyperglycemic conditions. Int. J. Biol. Sci..

[109] Richard D., Rivest R., Huang Q., Bouillaud F., Sanchis D., Champigny O., Ricquier D. (1998). Distribution of the uncoupling protein 2 mRNA in the mouse brain. J. Comp. Neurol..

[110] Rose G., Crocco P., De Rango F., Montesanto A., Passarino G. (2011). Further support to the uncoupling-to-survive theory: The genetic variation of human UCP genes is associated with longevity. PLoS ONE.

[111] Pecqueur C., Alves-Guerra C., Ricquier D., Bouillaud F. (2009). UCP2, a metabolic sensor coupling glucose oxidation to mitochondrial metabolism?. IUBMB Life.

[112] Pecqueur C., Bui T., Gelly C., Hauchard J., Barbot C., Bouillaud F., Ricquier D., Miroux B., Thompson C.B. (2008). Uncoupling protein-2 controls proliferation by promoting fatty acid oxidation and limiting glycolysis-derived pyruvate utilization. FASEB J..

[113] Echtay K.S., Roussel D., St-Pierre J., Jekabsons M.B., Cadenas S., Stuart J.A., Harper J.A., Roebuck S.J., Morrison A., Pickering S. (2002). Superoxide activates mitochondrial uncoupling proteins. Nature.

[114] Murphy M.P., Echtay K.S., Blaikie F.H., Asin-Cayuela J., Cocheme H.M., Green K., Buckingham J.A., Taylor E.R., Hurrell F., Hughes G. (2003). Superoxide activates uncoupling proteins by generating carbon-centered radicals and initiating lipid peroxidation: Studies using a mitochondria-targeted spin trap derived from alpha-phenyl-N-tert-butylnitrone. J. Biol. Chem..

[115] Beck V., Jaburek M., Demina T., Rupprecht A., Porter R.K., Jezek P., Pohl E.E. (2007). Polyunsaturated fatty acids activate human uncoupling proteins 1 and 2 in planar lipid bilayers. FASEB J..

[116] Gooley J.J. (2016). Circadian regulation of lipid metabolism. Proc. Nutr. Soc..

[117] Dickmeis T. (2009). Glucocorticoids and the circadian clock. J. Endocrinol..

[118] Harper M.E., Brand M.D. (1993). The quantitative contributions of mitochondrial proton leak and ATP turnover reactions to the changed respiration rates of hepatocytes from rats of different thyroid status. J. Biol. Chem..

[119] Harper M.E., Seifert E.L. (2008). Thyroid hormone effects on mitochondrial energetics. Thyroid.

[120] Ikegami K., Refetoff S., Van Cauter E., Yoshimura T. (2019). Interconnection between circadian clocks and thyroid function. Nat. Rev. Endocrinol..

[121] Vancamp P., Demeneix B.A. (2020). Is the observed decrease in body temperature during industrialization due to thyroid hormone-dependent thermoregulation disruption?. Front. Endocrinol..

[122] Russell W., Harrison R.F., Smith N., Darzy K., Shalet S., Weetman A.P., Ross R.J. (2008). Free triiodothyronine has a distinct circadian rhythm that is delayed but parallels thyrotropin levels. J. Clin. Endocrinol. Metab..

[123] Grivas T.B., Savvidou O.D. (2007). Melatonin the “light of night” in human biology and adolescent idiopathic scoliosis. Scoliosis.

[124] Tan D.X., Manchester L.C., Qin L., Reiter R.J. (2016). Melatonin: A mitochondrial targeting molecule involving mitochondrial protection and dynamics. Int. J. Mol. Sci..

[125] Gero D., Szabo C. (2016). Glucocorticoids suppress mitochondrial oxidant production via upregulation of uncoupling protein 2 in hyperglycemic endothelial cells. PLoS One.

[126] Hucklebridge F., Hussain T., Evans P., Clow A. (2005). The diurnal patterns of the adrenal steroids cortisol and dehydroepiandrosterone (DHEA) in relation to awakening. Psychoneuroendocrino.

[127] Galea A.M., Brown A.J. (2009). Special relationship between sterols and oxygen: Were sterols an adaptation to aerobic life?. Free Radic. Biol. Med..

[128] Lim L., Jackson-Lewis V., Wong L.C., Shui G.H., Goh A.X., Kesavapany S., Jenner A.M., Fivaz M., Przedborski S., Wenk M.R. (2012). Lanosterol induces mitochondrial uncoupling and protects dopaminergic neurons from cell death in a model for Parkinson’s disease. Cell Death Differ..

[129] Van Cauter E., Polonsky K.S., Scheen A.J. (1997). Roles of circadian rhythmicity and sleep in human glucose regulation. Endocr. Rev..

[130] Dulloo A.G., Gubler M., Montani J.P., Seydoux J., Solinas G. (2004). Substrate cycling between de novo lipogenesis and lipid oxidation: A thermogenic mechanism against skeletal muscle lipotoxicity and glucolipotoxicity. Int. J. Obes. Relat. Metab. Disord..

[131] Hiltunen J.K., Autio K.J., Schonauer M.S., Kursu V.A., Dieckmann C.L., Kastaniotis A.J. (2010). Mitochondrial fatty acid synthesis and respiration. Biochim. Biophys. Acta.

[132] Aviram R., Manella G., Kopelman N., Neufeld-Cohen A., Zwighaft Z., Elimelech M., Adamovich Y., Golik M., Wang C., Han X. (2016). Lipidomics analyses reveal temporal and spatial lipid organization and uncover daily oscillations in intracellular organelles. Mol. Cell.

[133] Randle P.J., Garland P.B., Hales C.N., Newsholme E.A. (1963). The glucose fatty-acid cycle. Its role in insulin sensitivity and the metabolic disturbances of diabetes mellitus. Lancet.

[134] Scheiermann C., Kunisaki Y., Frenette P.S. (2013). Circadian control of the immune system. Nat. Rev. Immunol..

[135] Lange T., Dimitrov S., Born J. (2010). Effects of sleep and circadian rhythm on the human immune system. Ann. N. Y. Acad. Sci..

[136] Raud B., McGuire P.J., Jones R.G., Sparwasser T., Berod L. (2018). Fatty acid metabolism in CD8^+^ T cell memory: Challenging current concepts. Immunol. Rev..

[137] Weinberg S.E., Chandel N.S. (2014). Futility sustains memory T cells. Immunity.

[138] Nowinski S.M., Solmonson A., Rusin S.F., Maschek J.A., Bensard C.L., Fogarty S., Jeong M.Y., Lettlova S., Berg J.A., Morgan J.T. (2020). Mitochondrial fatty acid synthesis coordinates oxidative metabolism in mammalian mitochondria. elife.

[139] Golbidi S., Badran M., Laher I. (2011). Diabetes and alpha lipoic Acid. Front. Pharmacol..

[140] Mailloux R.J. (2020). Protein S-glutathionylation reactions as a global inhibitor of cell metabolism for the desensitization of hydrogen peroxide signals. Redox Biol..

[141] Chretien D., Benit P., Ha H.H., Keipert S., El-Khoury R., Chang Y.T., Jastroch M., Jacobs H.T., Rustin P., Rak M. (2018). Mitochondria are physiologically maintained at close to 50 degrees C. PLoS Biol..

[142] Richardson R.B. (2022). The role of oxygen and the Goldilocks range in the development of cataracts induced by space radiation in US astronauts. Exp. Eye Res..

[143] Scialo F., Sriram A., Stefanatos R., Spriggs R.V., Loh S.H.Y., Martins L.M., Sanz A. (2020). Mitochondrial complex I derived ROS regulate stress adaptation in Drosophila melanogaster. Redox Biol..

[144] Sottile M.L., Nadin S.B. (2018). Heat shock proteins and DNA repair mechanisms: An updated overview. Cell Stress Chaperones.

[145] Zou J., Guo Y., Guettouche T., Smith D.F., Voellmy R. (1998). Repression of heat shock transcription factor HSF1 activation by HSP90 (HSP90 complex) that forms a stress-sensitive complex with HSF. Cell.

[146] Tsukamoto D., Hasegawa T., Hirose S.I., Sakurai Y., Ito M., Takamatsu N. (2019). Circadian transcription factor HSF1 regulates differential *HSP70* gene transcription during the arousal-torpor cycle in mammalian hibernation. Sci. Rep..

[147] Ohtsuka Y., Yabunaka N., Fujisawa H., Watanabe I., Agishi Y. (1994). Effect of thermal stress on glutathione metabolism in human erythrocytes. Eur. J. Appl. Physiol. Occup. Physiol..

[148] Slimen I.B., Najar T., Ghram A., Dabbebi H., Ben Mrad M., Abdrabbah M. (2014). Reactive oxygen species, heat stress and oxidative-induced mitochondrial damage. A review. Int. J. Hyperthermia.

[149] Hendriks K.D.W., Bruggenwirth I.M.A., Maassen H., Gerding A., Bakker B., Porte R.J., Henning R.H., Leuvenink H.G.D. (2019). Renal temperature reduction progressively favors mitochondrial ROS production over respiration in hypothermic kidney preservation. J. Transl. Med..

[150] Jarmuszkiewicz W., Woyda-Ploszczyca A., Koziel A., Majerczak J., Zoladz J.A. (2015). Temperature controls oxidative phosphorylation and reactive oxygen species production through uncoupling in rat skeletal muscle mitochondria. Free Radic. Biol. Med..

[151] Jones D.P., Sies H. (2015). The redox code. Antioxid. Redox Signal..

[152] Machado F.S.M., Zhang Z., Su Y., de Goede P., Jansen R., Foppen E., Coimbra C.C., Kalsbeek A. (2018). Time-of-day effects on metabolic and clock-related adjustments to cold. Front. Endocrinol..

[153] Brand M.D. (2000). Uncoupling to survive? The role of mitochondrial inefficiency in ageing. Exp. Gerontol..

[154] Herrero A., Barja G. (1998). H_2_O_2_ production of heart mitochondria and aging rate are slower in canaries and parakeets than in mice: Sites of free radical generation and mechanisms involved. Mech. Ageing Dev..

[155] Speakman J.R., Talbot D.A., Selman C., Snart S., McLaren J.S., Redman P., Krol E., Jackson D.M., Johnson M.S., Brand M.D. (2004). Uncoupled and surviving: Individual mice with high metabolism have greater mitochondrial uncoupling and live longer. Aging Cell.

[156] Cortassa S., O’Rourke B., Aon M.A. (2014). Redox-optimized ROS balance and the relationship between mitochondrial respiration and ROS. Biochim. Biophys. Acta.

[157] Marseglia L., D’Angelo G., Manti S., Arrigo T., Barberi I., Reiter R.J., Gitto E. (2014). Oxidative stress-mediated aging during the fetal and perinatal periods. Oxid. Med. Cell. Longev..

[158] Lodemore M., Petersen S.A., Wailoo M.P. (1991). Development of night time temperature rhythms over the first six months of life. Arch. Dis. Child.

[159] Brauner P., Kopecky P., Flachs P., Ruffer J., Sebron V., Plavka R., Vitkova I., Vorlicek J., Kopecky J. (2003). Induction of uncoupling protein 3 gene expression in skeletal muscle of preterm newborns. Pediatr. Res..

[160] Lean M.E., James W.P., Jennings G., Trayhurn P. (1986). Brown adipose tissue uncoupling protein content in human infants, children and adults. Clin. Sci..

[161] Piccione G., Caola G., Refinetti R. (2003). Daily and estrous rhythmicity of body temperature in domestic cattle. BMC Physiol..

[162] Bach V., Libert J.P. (2022). Hyperthermia and heat stress as risk factors for sudden infant death syndrome: A narrative review. Front. Pediatr..

[163] Yates J. (2018). Perspective: The long-term effects of light exposure on establishment of newborn circadian rhythm. J. Clin. Sleep Med..

[164] Harrington C.T., Hafid N.A., Waters K.A. (2022). Butyrylcholinesterase is a potential biomarker for Sudden Infant Death Syndrome. EBioMedicine.

[165] Haynes R.L., Frelinger A.L., Giles E.K., Goldstein R.D., Tran H., Kozakewich H.P., Haas E.A., Gerrits A.J., Mena O.J., Trachtenberg F.L. (2017). High serum serotonin in sudden infant death syndrome. Proc. Natl. Acad. Sci. USA.

[166] Shekhawat P.S., Matern D., Strauss A.W. (2005). Fetal fatty acid oxidation disorders, their effect on maternal health and neonatal outcome: Impact of expanded newborn screening on their diagnosis and management. Pediatr. Res..

[167] Maher P. (2005). The effects of stress and aging on glutathione metabolism. Ageing Res. Rev..

[168] Aiello A., Farzaneh F., Candore G., Caruso C., Davinelli S., Gambino C.M., Ligotti M.E., Zareian N., Accardi G. (2019). Immunosenescence and its hallmarks: How to oppose aging strategically? A review of potential options for therapeutic intervention. Front. Immunol..

[169] Millyard A., Layden J.D., Pyne D.B., Edwards A.M., Bloxham S.R. (2020). Impairments to thermoregulation in the elderly during heat exposure events. Gerontol. Geriatr. Med..

[170] Waalen J., Buxbaum J.N. (2011). Is older colder or colder older? The association of age with body temperature in 18,630 individuals. J. Gerontol. Biol. Sci. Med. Sci..

[171] Eggenberger P., Burgisser M., Rossi R.M., Annaheim S. (2021). Body temperature is associated with cognitive performance in older adults with and without mild cognitive impairment: A cross-sectional analysis. Front. Aging Neurosci..

[172] Choromanska B., Mysliwiec P., Luba M., Wojskowicz P., Mysliwiec H., Choromanska K., Dadan J., Zalewska A., Maciejczyk M. (2020). The impact of hypertension and metabolic syndrome on nitrosative stress and glutathione metabolism in patients with morbid obesity. Oxid. Med. Cell. Longev..

[173] Sabens Liedhegner E.A., Gao X.H., Mieyal J.J. (2012). Mechanisms of altered redox regulation in neurodegenerative diseases—Focus on S-glutathionylation. Antioxid. Redox Signal..

[174] Cha S.J., Kim H., Choi H.J., Lee S., Kim K. (2017). Protein glutathionylation in the pathogenesis of neurodegenerative diseases. Oxid. Med. Cell. Longev..

[175] Mingrone G., Rosa G., Greco A.V., Manco M., Vega N., Hesselink M.K., Castagneto M., Schrauwen P., Vidal H. (2003). Decreased uncoupling protein expression and intramyocytic triglyceride depletion in formerly obese subjects. Obes. Res..

[176] Shinozaki S., Chang K., Sakai M., Shimizu N., Yamada M., Tanaka T., Nakazawa H., Ichinose F., Yamada Y., Ishigami A. (2014). Inflammatory stimuli induce inhibitory S-nitrosylation of the deacetylase SIRT1 to increase acetylation and activation of p53 and p65. Sci. Signal.

[177] Hood S., Amir S. (2017). Neurodegeneration and the circadian clock. Front Aging Neurosci.

[178] Vrettou S., Wirth B. (2022). S-glutathionylation and S-nitrosylation in mitochondria: Focus on homeostasis and neurodegenerative diseases. Int. J. Mol. Sci..

[179] Karhu T., Myllymaa S., Nikkonen S., Mazzotti D.R., Toyras J., Leppanen T. (2021). Longer and deeper desaturations are associated with the worsening of mild sleep apnea: The Sleep Heart Health Study. Front. Neurosci..

[180] Lacedonia D., Carpagnano G.E., Crisetti E., Cotugno G., Palladino G.P., Patricelli G., Sabato R., Foschino Barbaro M.P. (2015). Mitochondrial DNA alteration in obstructive sleep apnea. Respir. Res..

[181] Demine S., Renard P., Arnould T. (2019). Mitochondrial uncoupling: A key controller of biological processes in physiology and diseases. Cells.

[182] Rogers R.S., Morris E.M., Wheatley J.L., Archer A.E., McCoin C.S., White K.S., Wilson D.R., Meers G.M., Koch L.G., Britton S.L. (2016). Deficiency in the heat stress response could underlie susceptibility to metabolic disease. Diabetes.

[183] Koziel A., Sobieraj I., Jarmuszkiewicz W. (2015). Increased activity of mitochondrial uncoupling protein 2 improves stress resistance in cultured endothelial cells exposed in vitro to high glucose levels. Am. J. Physiol. Heart Circ. Physiol..

[184] Sreedhar A., Cassell T., Smith P., Lu D., Nam H.W., Lane A.N., Zhao Y. (2019). UCP2 overexpression redirects glucose into anabolic metabolic pathways. Proteomics.

[185] Twig G., Shirihai O.S. (2011). The interplay between mitochondrial dynamics and mitophagy. Antioxid. Redox Signal..

[186] Cadenas S. (2018). Mitochondrial uncoupling, ROS generation and cardioprotection. Biochim. Biophys. Acta. Bioenerg..

[187] Leak R.K. (2014). Heat shock proteins in neurodegenerative disorders and aging. J. Cell. Commun. Signal..

[188] Leng Y., Musiek E.S., Hu K., Cappuccio F.P., Yaffe K. (2019). Association between circadian rhythms and neurodegenerative diseases. Lancet Neurol..

[189] Sulli G., Lam M.T.Y., Panda S. (2019). Interplay between circadian clock and cancer: New frontiers for cancer treatment. Trends Cancer.

[190] Zimmet P., Alberti K., Stern N., Bilu C., El-Osta A., Einat H., Kronfeld-Schor N. (2019). The circadian syndrome: Is the metabolic syndrome and much more!. J. Intern. Med..

[191] Aguilar-Lopez B.A., Moreno-Altamirano M.M.B., Dockrell H.M., Duchen M.R., Sanchez-Garcia F.J. (2020). Mitochondria: An integrative hub coordinating circadian rhythms, metabolism, the microbiome, and immunity. Front. Cell Dev. Biol..

[192] Pietroiusti A., Neri A., Somma G., Coppeta L., Iavicoli I., Bergamaschi A., Magrini A. (2010). Incidence of metabolic syndrome among night-shift healthcare workers. Occup. Environ. Med..

[193] Walker W.H., Borniger J.C. (2019). Molecular mechanisms of cancer-induced sleep disruption. Int. J. Mol. Sci..

[194] Bevinakoppamath S., Ramachandra S.C., Yadav A.K., Basavaraj V., Vishwanath P., Prashant A. (2021). Understanding the emerging link between circadian rhythm, Nrf2 pathway, and breast cancer to overcome drug resistance. Front. Pharmacol..

[195] Koritala B.S.C., Porter K.I., Arshad O.A., Gajula R.P., Mitchell H.D., Arman T., Manjanatha M.G., Teeguarden J., Van Dongen H.P.A., McDermott J.E. (2021). Night shift schedule causes circadian dysregulation of DNA repair genes and elevated DNA damage in humans. J. Pineal Res..

[196] Richardson R.B., Anghel C.V., Deng D.S. (2021). Profound synchrony of age-specific incidence rates and tumor suppression for different cancer types as revealed by the multistage-senescence model of carcinogenesis. Aging.

[197] Van Cauter E. (1990). Diurnal and ultradian rhythms in human endocrine function: A minireview. Horm. Res..

[198] Tocchetti C.G., Stanley B.A., Sivakumaran V., Bedja D., O’Rourke B., Paolocci N., Cortassa S., Aon M.A. (2015). Impaired mitochondrial energy supply coupled to increased H_2_O_2_ emission under energy/redox stress leads to myocardial dysfunction during Type I diabetes. Clin. Sci..

[199] Rains J.L., Jain S.K. (2011). Oxidative stress, insulin signaling, and diabetes. Free Radic. Biol. Med..

[200] Harding E.C., Franks N.P., Wisden W. (2019). The temperature dependence of sleep. Front. Neurosci..

[201] Altinoz M.A., Ozpinar A., Seyfried T.N. (2020). Caprylic (octanoic) acid as a potential fatty acid chemotherapeutic for glioblastoma. Prostaglandins Leukot. Essent. Fatty Acids.

[202] Carey H.V., Rhoads C.A., Aw T.Y. (2003). Hibernation induces glutathione redox imbalance in ground squirrel intestine. J. Comp. Physiol. B.

[203] Barger J.L., Barnes B.M., Boyer B.B. (2006). Regulation of UCP1 and UCP3 in Arctic ground squirrels and relation with mitochondrial proton leak. J. Appl. Physiol..

[204] Boyer B.B., Barnes B.M. (1999). Molecular and metabolic aspects of mammal hibernation. Bioscience.

[205] Brown J.C., Chung D.J., Belgrave K.R., Staples J.F. (2012). Mitochondrial metabolic suppression and reactive oxygen species production in liver and skeletal muscle of hibernating thirteen-lined ground squirrels. Am. J. Physiol. Regul. Integr. Comp. Physiol..

[206] Watts A.J., Storey K.B. (2022). Peripheral circadian gene activity is altered during hibernation in the thirteen-lined ground squirrel. Cryobiology.

[207] Turbill C., Bieber C., Ruf T. (2011). Hibernation is associated with increased survival and the evolution of slow life histories among mammals. Proc. Biol. Sci..

[208] Strollo F., Gentile S., Strollo G., Mambro A., Vernikos J. (2018). Recent progress in space physiology and aging. Front. Physiol..

[209] Richardson R.B., Harper M.E. (2016). Mitochondrial stress controls the radiosensitivity of the oxygen effect: Implications for radiotherapy. Oncotarget.

[210] Scrima R., Cela O., Agriesti F., Piccoli C., Tataranni T., Pacelli C., Mazzoccoli G., Capitanio N. (2020). Mitochondrial calcium drives clock gene-dependent activation of pyruvate dehydrogenase and of oxidative phosphorylation. Biochim. Biophys. Acta Mol. Cell Res..

[211] Bisserier M., Shanmughapriya S., Rai A.K., Gonzalez C., Brojakowska A., Garikipati V.N.S., Madesh M., Mills P.J., Walsh K., Arakelyan A. (2021). Cell-free mitochondrial DNA as a potential biomarker for astronauts’ health. J. Am. Heart Assoc..

[212] da Silveira W.A., Fazelinia H., Rosenthal S.B., Laiakis E.C., Kim M.S., Meydan C., Kidane Y., Rathi K.S., Smith S.M., Stear B. (2020). Comprehensive multi-omics analysis reveals mitochondrial stress as a central biological hub for spaceflight impact. Cell.

[213] Pecaut M.J., Mao X.W., Bellinger D.L., Jonscher K.R., Stodieck L.S., Ferguson V.L., Bateman T.A., Mohney R.P., Gridley D.S. (2017). Is spaceflight-induced immune dysfunction linked to systemic changes in metabolism?. PLoS ONE.

[214] Navarro J., Obrador E., Pellicer J.A., Aseni M., Vina J., Estrela J.M. (1997). Blood glutathione as an index of radiation-induced oxidative stress in mice and humans. Free Radic. Biol. Med..

[215] Stahn A.C., Werner A., Opatz O., Maggioni M.A., Steinach M., von Ahlefeld V.W., Moore A., Crucian B.E., Smith S.M., Zwart S.R. (2017). Increased core body temperature in astronauts during long-duration space missions. Sci. Rep..

[216] Patten D.A., Ouellet M., Allan D.S., Germain M., Baird S.D., Harper M.E., Richardson R.B. (2019). Mitochondrial adaptation in human mesenchymal stem cells following ionizing radiation. FASEB J..

[217] Speakman J.R., Selman C., McLaren J.S., Harper E.J. (2002). Living fast, dying when? The link between aging and energetics. J. Nutr..

[218] Buttgereit F., Brand M.D. (1995). A hierarchy of ATP-consuming processes in mammalian cells. Biochem. J..

[219] Petrushanko I.Y., Yakushev S., Mitkevich V.A., Kamanina Y.V., Ziganshin R.H., Meng X., Anashkina A.A., Makhro A., Lopina O.D., Gassmann M. (2012). S-Glutathionylation of the Na,K-ATPase catalytic α subunit is a determinant of the enzyme redox sensitivity. J. Biol. Chem..

[220] Bilen M., Benhammouda S., Slack R.S., Germain M. (2022). The integrated stress response as a key pathway downstream of mitochondrial dysfunction. Curr. Opin. Physiol..

[221] Estrela J.M., Pallardo F.V., Viña J. (1990). Role of glutathione in the regulation of protein synthesis and degradation in eukaryotes. Gluathione: Metabolism and Physiological Functions.

[222] Patten D.A., McGuirk S., Anilkumar U., Antoun G., Gandhi K., Parmar G., Iqbal M.A., Wong J., Richardson R.B., St-Pierre J. (2021). Altered mitochondrial fusion drives defensive glutathione synthesis in cells able to switch to glycolytic ATP production. Biochim. Biophys. Acta Mol. Cell Res..

[223] Reimund E. (1994). The free radical flux theory of sleep. Med. Hypotheses.

[224] Eugene A.R., Masiak J. (2015). The neuroprotective aspects of sleep. MEDtube Sci..

[225] Xie L., Kang H., Xu Q., Chen M.J., Liao Y., Thiyagarajan M., O’Donnell J., Christensen D.J., Nicholson C., Iliff J.J. (2013). Sleep drives metabolite clearance from the adult brain. Science.

[226] Piéron H. (1912). Le Problème Physiologique du Sommeil.

[227] Komoda Y., Honda K., Inoue S. (1990). SPS-B, a physiological sleep regulator, from the brainstems of sleep-deprived rats, identified as oxidized glutathione. Chem. Pharm Bull..

[228] Adolfsen K.J., Brynildsen M.P. (2015). Futile cycling increases sensitivity toward oxidative stress in *Escherichia coli*. Metab. Eng..

[229] Asher G., Reinke H., Altmeyer M., Gutierrez-Arcelus M., Hottiger M.O., Schibler U. (2010). Poly(ADP-ribose) polymerase 1 participates in the phase entrainment of circadian clocks to feeding. Cell.

[230] Pietrzak J., Spickett C.M., Ploszaj T., Virag L., Robaszkiewicz A. (2018). PARP1 promoter links cell cycle progression with adaptation to oxidative environment. Redox Biol..

[231] Feillet C., van der Horst G.T., Levi F., Rand D.A., Delaunay F. (2015). Coupling between the circadian clock and cell cycle oscillators: Implication for healthy cells and malignant growth. Front. Neurol..

[232] Yousefzadeh M., Henpita C., Vyas R., Soto-Palma C., Robbins P., Niedernhofer L. (2021). DNA damage-how and why we age?. elife.

[233] Kang T.H., Lindsey-Boltz L.A., Reardon J.T., Sancar A. (2010). Circadian control of XPA and excision repair of cisplatin-DNA damage by cryptochrome and HERC2 ubiquitin ligase. Proc. Natl. Acad. Sci. USA.

[234] Kang T.H. (2021). Circadian rhythm of NER and ATR pathways. Biomolecules.

[235] Manzella N., Bracci M., Strafella E., Staffolani S., Ciarapica V., Copertaro A., Rapisarda V., Ledda C., Amati M., Valentino M. (2015). Circadian modulation of 8-oxoguanine DNA damage repair. Sci. Rep..

[236] Richardson R.B. (2009). Ionizing radiation and aging: Rejuvenating an old idea. Aging.

[237] Zada D., Sela Y., Matosevich N., Monsonego A., Lerer-Goldshtein T., Nir Y., Appelbaum L. (2021). Parp1 promotes sleep, which enhances DNA repair in neurons. Mol. Cell.

[238] Rong Z., Tu P., Xu P., Sun Y., Yu F., Tu N., Guo L., Yang Y. (2021). The mitochondrial response to DNA damage. Front. Cell Dev. Biol..

[239] Rabinovich-Nikitin I., Lieberman B., Martino T.A., Kirshenbaum L.A. (2019). Circadian-regulated cell death in cardiovascular diseases. Circulation.

[240] Shutt T., Geoffrion M., Milne R., McBride H.M. (2012). The intracellular redox state is a core determinant of mitochondrial fusion. EMBO Rep..

[241] Yang Z., Wang L., Yang C., Pu S., Guo Z., Wu Q., Zhou Z., Zhao H. (2021). Mitochondrial membrane remodeling. Front. Bioeng. Biotechnol..

[242] Sancar A., Lindsey-Boltz L.A., Kang T.H., Reardon J.T., Lee J.H., Ozturk N. (2010). Circadian clock control of the cellular response to DNA damage. FEBS Lett..

[243] Kempf A., Song S.M., Talbot C.B., Miesenbock G. (2019). A potassium channel beta-subunit couples mitochondrial electron transport to sleep. Nature.

[244] Andreazza A.C., Andersen M.L., Alvarenga T.A., de-Oliveira M.R., Armani F., Ruiz F.S., Giglio L., Moreira J.C., Kapczinski F., Tufik S. (2010). Impairment of the mitochondrial electron transport chain due to sleep deprivation in mice. J. Psychiatr. Res..

[245] Cirelli C., Tononi G. (2004). Uncoupling proteins and sleep deprivation. Arch. Ital. Biol..

[246] Bar-Yaacov D., Blumberg A., Mishmar D. (2012). Mitochondrial-nuclear co-evolution and its effects on OXPHOS activity and regulation. Biochim. Biophys. Acta.

